# A Living Multi‐Omics PDX Biobank of High‐Risk Sarcomas Reveals ecDNA‐Driven Oncogene Amplification in Osteosarcoma and Enables Preclinical Precision Oncology

**DOI:** 10.1002/advs.78200

**Published:** 2026-10-12

**Authors:** Weisong Zhao, Zhiwei Cheng, Kai Tian, Mengxiong Sun, Zhuoying Wang, Ming Jiao, Jens Luebeck, Kaiyuan Zhu, Xiao Zhang, Dongqing Zuo, Hongsheng Wang, Chongren Wang, Jiakang Shen, Xiaojun Ma, Gangyang Wang, Zongyi Wang, Jing Han, Kaiyuan Liu, Haoran Mu, Weiqi Ma, Yongyong Ren, Shibing Guo, Huangxin Tu, Lingfeng Yu, Yining Tao, Yu Lv, Hui Lu, Owen S. Chapman, Rishaan Kenkre, Lukas Chavez, Zhengdong Cai, Yuqin Yang, Wei Sun, Yafei Jiang, Yingqi Hua

**Affiliations:** ^1^ Department of Orthopaedics Shanghai General Hospital Shanghai Jiao Tong University School of Medicine Shanghai People's Republic of China; ^2^ Shanghai Bone Tumor Institution Shanghai People's Republic of China; ^3^ SJTU‐Yale Joint Center of Biostatistics and Data Science National Center for Translational Medicine Shanghai Jiao Tong University Shanghai People's Republic of China; ^4^ Department of Laboratory Animal Center Shanghai General Hospital Shanghai Jiao Tong University School of Medicine Shanghai People's Republic of China; ^5^ Department of Computer Science and Engineering University of California San Diego California USA; ^6^ School of Computer Science Shanghai Jiao Tong University Shanghai People's Republic of China; ^7^ Department of Bioinformatics and Biostatistics School of Life Sciences and Biotechnology Shanghai Jiao Tong University Shanghai People's Republic of China; ^8^ Department of Orthopedic Surgery Second Affiliated Hospital of Inner Mongolia Medical University Inner Mongolia People's Republic of China; ^9^ Department of Neuro‐oncology Nagoya City University Graduate School of Medical Sciences Nagoya Japan; ^10^ Sanford Burnham Prebys Medical Discovery Institute La Jolla California USA; ^11^ Department of Medicine University of California San Diego La Jolla California USA; ^12^ Rady Children's Institute for Genomic Medicine Rady Children's Hospital and Healthcare Center San Diego California USA

## Abstract

Sarcomas are rare, genomically heterogeneous malignancies for which prognosis has remained poor and largely unchanged over recent decades. Patient‐derived xenograft (PDX) models are an important tool for cancer research, but their availability and utility remain limited in sarcoma. Here, we establish the SGH sarcoma PDX biobank, a living multi‐omics resource comprising 155 xenografts from 132 patients across 11 sarcoma histotypes, with osteosarcoma as the predominant subtype. This cohort is enriched for high‐risk sarcomas characterized by poor prognosis and widespread copy‐number alterations. Using this biobank, we show that extrachromosomal DNA (ecDNA) frequently drives high‐level oncogene amplification in osteosarcoma and that *MYC* ecDNA may underlie aggressive disease trajectories. Analyses of paired patient and PDX samples demonstrate that ecDNA is preferentially retained over intrachromosomal amplicons during engraftment and is stably maintained in PDX models. In addition, the PDX models largely recapitulate the histopathological and molecular features of their matched patient tumors while exhibiting distinct clonal evolutionary trajectories during engraftment. Finally, we identified potentially actionable genomic alterations within this PDX cohort, demonstrated genotype‐matched targeted therapy in a KRAS G12C‐mutant PDX model, and prioritized several candidate antibody–drug conjugate targets. Together, this resource provides a platform for biological discovery and preclinical precision oncology for sarcoma.

## Graphical abstract

A living multi‐omics sarcoma PDX biobank is enriched for high‐risk disease and preserves key patient tumor features at histopathological and molecular levels. Integrated profiling reveals ecDNA‐driven oncogene amplification in osteosarcoma and preferential retention of ecDNA during PDX evolution. This resource enables biological discovery and preclinical therapeutic evaluation.

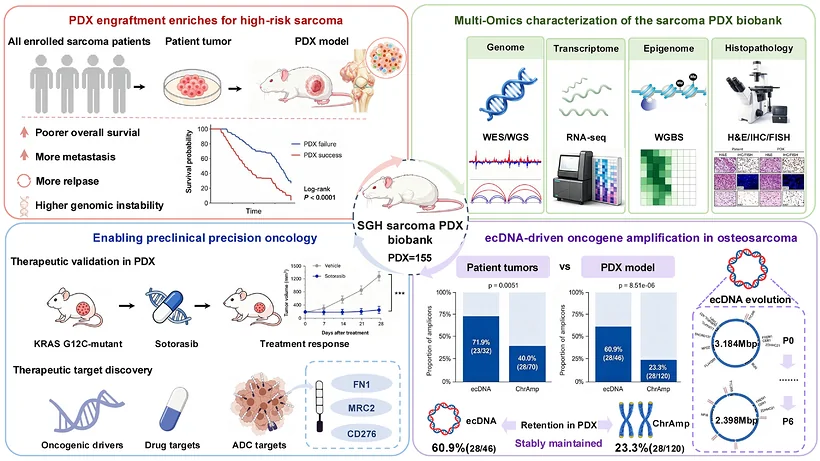

## 1 Introduction

Sarcomas are rare, heterogeneous malignancies of mesenchymal origin that arise predominantly in bone and soft tissue, encompassing over 70 histological subtypes [1, 2]. Although they represent only ∼1% of adult cancers, sarcomas are relatively more common in children, accounting for ∼20% of non‐hematologic malignancies in the pediatric population [3]. Over the past three decades, progress in sarcoma treatment has largely stagnated, and 5‐year overall survival (OS) has remained at 50%–60% for bone sarcomas and ∼65% for soft tissue sarcomas [4, 5]. Outcomes are particularly poor in metastatic sarcoma, with 5‐year OS in metastatic osteosarcoma of only ∼20%–30% [6, 7]. Sarcomas often exhibit highly complex genomes with frequent chromothripsis [8, 9]. However, their genomic architecture varies substantially by subtype, ranging from relatively simple karyotypes driven by recurrent fusion oncogenes in Ewing sarcoma to highly complex karyotypes with extensive chromosomal rearrangements and copy‐number alterations (CNAs) in osteosarcoma [10, 11, 12].

The advent of next‐generation sequencing (NGS) has enabled precision oncology, where treatment decisions are guided by tumor‐specific molecular alterations rather than histology alone [13]. In a large, targeted panel sequencing study of 7,494 sarcomas representing 44 histological subtypes, potentially actionable genetic alterations were identified in 31.7% of tumors [14]. Similarly, analysis of whole‐genome sequencing (WGS) data from the 100,000 Genomes Project (Genomics England), including 1,617 patients with sarcoma, identified actionable alterations in 34% of cases [15]. Furthermore, molecular subtyping has become a key precision oncology strategy in tumors such as medulloblastoma [16], breast cancer [17], and small‐cell lung cancer [18], where distinct molecular subtypes guide subtype‐directed therapy. Our previous multi‐omics analysis of osteosarcoma identified four molecular subtypes with distinct biological features and clinical outcomes, including a high‐risk *MYC*‐driven subtype with the poorest prognosis [19]. Proteomic studies in other sarcomas have also defined clinically relevant subgroups that support risk stratification and therapy selection [20, 21]. However, these molecularly targeted and subtype‐informed strategies still require rigorous validation in faithful preclinical models before they can be translated into clinical practice.

Patient‐derived xenograft (PDX) models, generated by engrafting patient tumor tissue into immunodeficient mice, are widely used in cancer research [22]. Compared with established cancer cell lines, PDX models more faithfully recapitulate the histopathological and genomic features of human tumors, as well as intratumoral heterogeneity [22, 23]. However, currently available sarcoma PDX models are few compared with those for other adult malignancies and are predominantly derived from pediatric patients [24, 25, 26, 27], thereby providing only a limited representation of sarcoma biology. This limitation is particularly pronounced for osteosarcoma, which has a highly complex and heterogeneous genome [24, 28, 29]. In this study, we established the Shanghai General Hospital (SGH) sarcoma PDX biobank, a living multi‐omics resource comprising 155 models from 132 patients across 11 sarcoma histotypes, with osteosarcoma predominating. Clinical and molecular analyses indicate that PDX engraftment is enriched for biologically aggressive, high‐risk disease, highlighting the value of this resource. To our knowledge, this is the largest multi‐omics sarcoma PDX collection established from a Chinese patient population. Here, we provide this resource to the research community to enable deeper studies of sarcoma genomics and to support preclinical evaluation of candidate therapeutics.

## 2 Results

### 2.1 Establishment of the Living Multi‐Omics SGH Sarcoma PDX Biobank

We launched the SGH Sarcoma PDX Project in 2014, aiming to build a comprehensive sarcoma PDX biobank with a focus on osteosarcoma. Fresh tumor specimens from patients at Shanghai General Hospital (China) were subcutaneously implanted into NOD scid gamma (NSG) mice to generate PDXs. By January 2024, engraftment had been attempted for 351 tumors from 313 patients (Figure S1A,B). The overall engraftment rate was 44.2%, with synovial sarcoma showing the highest success rate at 56.3% and chondrosarcoma the lowest at 25% (Table S1). In osteosarcoma, the engraftment rate was 44.9%, which was lower than the 67% previously reported for subcutaneous PDX models [27]. In total, we established 155 PDX models from 132 patients across 11 sarcoma histotypes, with osteosarcoma predominating (Figure 1A). The cohort had a male‐to‐female ratio of 1.4:1 and a median age of 23 years (interquartile range [IQR 14‐44]). Clinical annotation showed that most models were derived from resection specimens (147/155, 94.8%) and spanned a broad range of tumor sites and disease stages, including 81 (52.2%) from primary tumors, 30 (19.4%) from metastatic tumors, and 44 (28.4%) from recurrent tumors (Figure 1B; Table S1).

**FIGURE 1:**
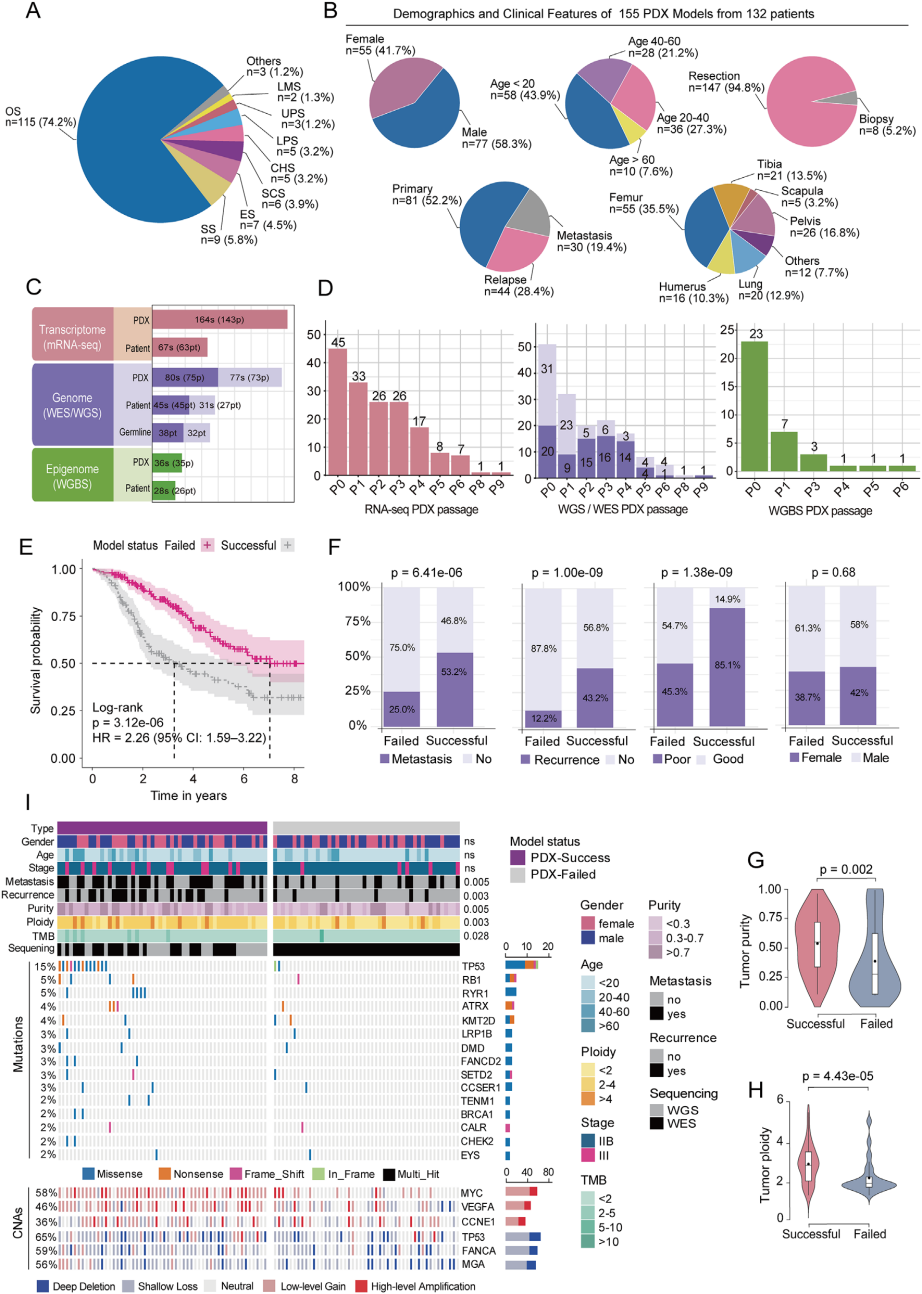
Establishing the SGH Sarcoma PDX Biobank. (A) Distribution of tumor histotypes among 155 successfully established PDX models derived from 132 patients. (B) Demographic and clinical characteristics of these patients, including sex, age group, anatomic tumor site, specimen type, and disease status (primary, metastatic, or recurrent). (C) Overview of multi‐omics datasets generated in this study; “s” denotes samples, and “p” and “pt” denote PDXs and patients, respectively. (D) Distribution of PDX passages profiled by RNA‐seq, whole exome sequencing/whole genome sequencing (WES/WGS), and whole‐genome bisulfite sequencing (WGBS) across the cohort; dark purple denotes WGS and light purple denotes WES. (E) Kaplan–Meier analysis of overall survival in 313 patients with successful (*n* = 132) versus failed (*n* = 181) PDX engraftment; the *p‐*value was calculated using a log‐rank test. (F) Clinical characteristics in patients with successful versus failed PDX engraftment, including metastasis, recurrence, chemotherapy response, and sex; *p*‐values were calculated using two‐sided chi‐square tests. (G–H) Comparison of (G) tumor purity and (H) ploidy between 54 osteosarcoma patients with successful PDX engraftment and 48 with failed engraftment; *p*‐values were calculated using two‐sided Wilcoxon rank‐sum tests. (I) Integrated clinical and genomic landscape of osteosarcoma in 54 patients with PDX engraftment and 48 without, showing clinical annotations (top), mutations (middle), and copy number alterations (bottom); *p*‐values were calculated using Fisher's exact test and Wilcoxon rank‐sum tests with “ns” indicating *p* ≥ 0.05. Abbreviations: OS, osteosarcoma; SS, synovial sarcoma; ES, Ewing sarcoma; SCS, spindle cell sarcoma; CHS, chondrosarcoma; LPS, liposarcoma; PS, pleomorphic sarcoma; LMS, leiomyosarcoma. “Others” include CB (chondroblastoma), GCTB (giant cell tumor of bone), and RMS (rhabdomyosarcoma).

DNA and RNA sequencing were performed for each PDX model where feasible. After quality control filtering, RNA‐seq data were available for 143 PDXs and DNA sequencing data for 148 PDXs (Figure 1C). Corresponding patient tumors provided RNA‐seq data from 67 samples and DNA sequencing data from 76 samples. We also performed whole‐genome bisulfite sequencing (WGBS) on 35 osteosarcoma PDXs and 28 patient tumors. PDX sequencing spanned multiple passages, with most data derived from early passages (Figure 1D). In addition, we established 42 longitudinal PDX models from 19 patients, derived from tumor specimens collected at distinct clinical disease states within the same individual, enhancing representation of disease progression across the cohort (Figure S1C). Detailed clinical and sequencing information for each PDX model is provided in Table S2.

### 2.2 PDX Engraftment Identifies High‐Risk Sarcoma Patients

We evaluated potential patient selection bias associated with PDX engraftment by comparing patients whose tumors successfully engrafted and patients whose tumors did not engraft. Survival analysis showed that patients with engraftment had significantly worse OS than those without (hazard ratio [HR] 2.26; log‐rank *p* = 3.12e‐06; Figure 1E). Clinical analysis indicated that successful engraftment was significantly associated with higher rates of metastasis, recurrence, and poor chemotherapy response, with no difference by sex, consistent with a more aggressive disease phenotype among patients whose tumors engrafted (Figure 1F).

To investigate genomic characteristics associated with engraftment, we analyzed paired tumor and normal DNA sequencing data from 102 patients with osteosarcoma, including 54 with engraftment and 48 without. Patients with engraftment exhibited higher tumor purity and ploidy (Figure 1G,H). The overall TP53 mutation rate was 15% (Figure 1I), similar to our prior report of 11% [19], but TP53 mutations were enriched in the engraftment group (24.1% vs. 4.2%; Fisher's exact test, *p* = 0.0049), suggesting greater genomic instability in this group. In addition, patients with engraftment had a greater CNA burden, most notably *MYC* gain/amplification (83.3% vs. 29.2%; Fisher's exact test, *p* = 2.96e‐08; Figure 1I). Using available RNA‐seq data, we observed significant activation of *MYC* target gene sets and other proliferation‐related pathways in the engraftment group (Figure S1D). These findings, consistent with our previous study [19] and other reports [30, 31], highlight the role of *MYC* in osteosarcoma aggressiveness. Collectively, these data also reveal that successful PDX engraftment enriches for biologically aggressive disease and represents a clinically and genomically high‐risk sarcoma subset.

### 2.3 Genomic Landscape of the SGH Sarcoma PDX Cohort

A total of 148 unique PDX models from 126 patients were included in the genomic landscape analysis, 110 of which were osteosarcoma (74.3%; Figure 2A). Across the cohort, we identified 13,752 somatic mutations, comprising 13,033 single‐nucleotide variants (SNVs) and 719 insertions or deletions (INDELs). The tumor mutational burden was low (median 1.28 mutations per megabase), consistent with prior sarcoma studies [32, 33]. MutSigCV identified *TP53* as the only significantly mutated driver (26%), with additional recurrent mutations in *LRP1B* (9%), *RYR1* (9%), *DMD* (6%), and *RB1* (6%) (Figure 2B; Table S3). *TP53* mutations were more frequent in osteosarcoma than in other sarcomas (30.9% vs. 13.2%; Fisher's exact test *p* = 0.034; Figure S2B). Using non‐negative matrix factorization, we deconvoluted somatic mutation profiles into three mutational signatures (Sig1 to Sig3; Figure S2C). Sig1 showed the highest cosine similarity to COSMIC SBS6 (defective DNA mismatch repair), Sig2 matched SBS35 (prior platinum exposure), and Sig3 resembled SBS4 (tobacco‐associated processes). Sig1 and Sig2 predominated in osteosarcoma PDXs, consistent with the pronounced genomic instability of osteosarcoma and the routine use of platinum‐based neoadjuvant therapy.

**FIGURE 2:**
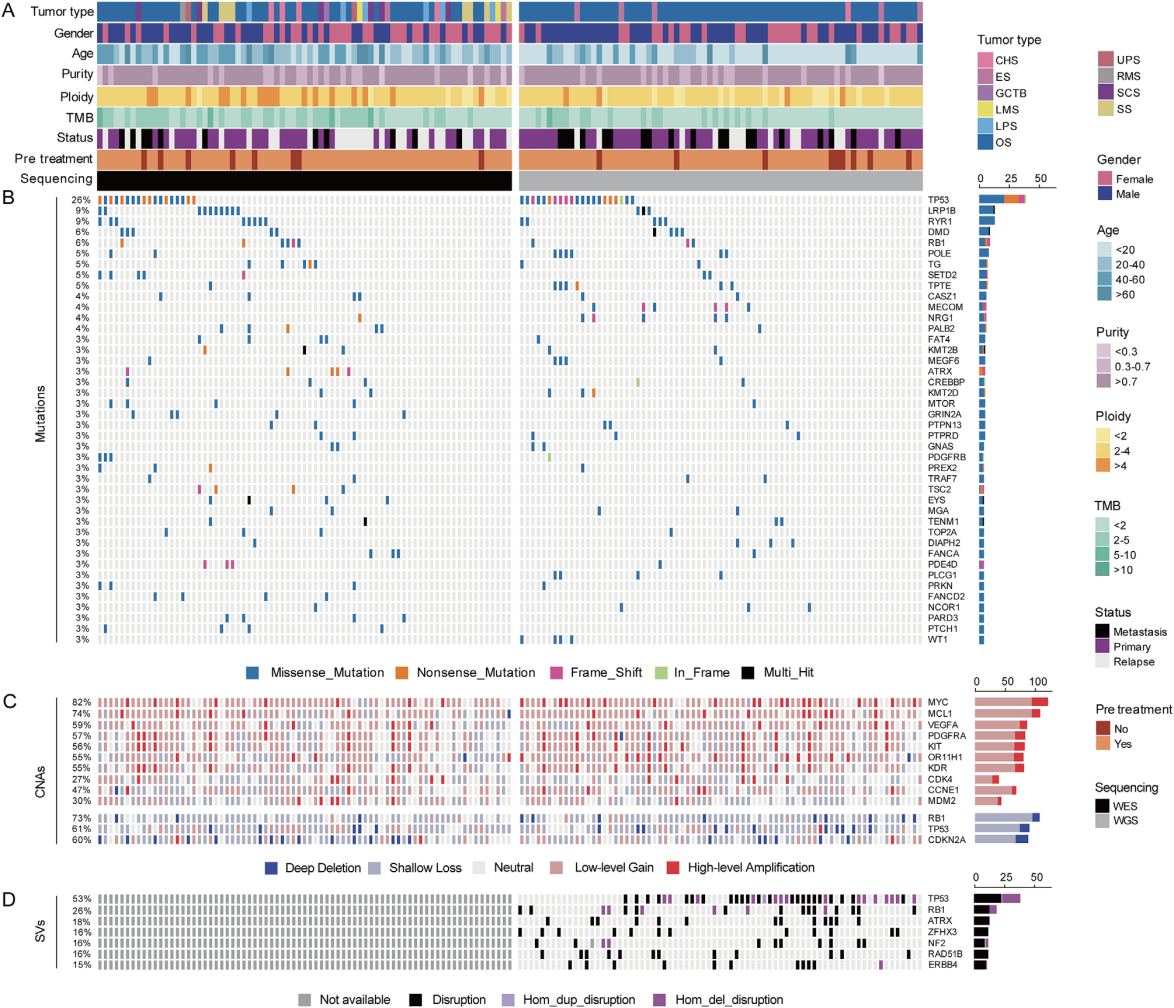
Genomic Landscape of the SGH Sarcoma PDX Cohort. (A) Clinical and genomic annotations for 148 unique PDX models; each column represents one PDX and is annotated for tumor type, sex, age group, tumor purity, ploidy category, tumor mutational burden (TMB) category, disease status (primary, metastatic, or relapse), pretreatment status, and sequencing type (WES or WGS). (B) Recurrently mutated driver genes (present in >2% of PDX models), with mutation class indicated by color (missense, nonsense, frameshift, in‐frame, and multi‐hit). (C) Somatic copy number alterations affecting driver genes located in significant GISTIC peaks and showing high levels of amplification or deletion in >10 PDX models. *CDK4*, *CCNE1*, and *MDM2* were manually added because they are well‐established oncogenes in sarcomas. Copy number states are shown as high‐level amplification (+2), low‐level gain (+1), neutral (0), shallow loss (−1), and deep deletion (−2). (D) Structural variants (SVs) involving driver genes that are recurrently disrupted in WGS‐sequenced PDX models (>10 PDX models), including LINX‐annotated disruption drivers (DISRUPTION) and homozygous disruption drivers (HOM_DUP_DISRUPTION and HOM_DEL_DISRUPTION).

We next examined oncogenes and tumor suppressor genes significantly affected by somatic CNAs across the cohort (Figure 2C; Table S4). *MYC* (8q24.21) showed the highest frequency of gain (82%), with additional recurrent gains at *MCL1* (1q21.2, 74%), *VEGFA* (6p12.1, 59%), *PDGFRA* (4q12, 57%), and *KIT* (4q12, 56%). In contrast, losses were frequent in tumor suppressor genes, including *RB1* (13q14.2, 73%), *TP53* (17p13.1, 61%), and *CDKN2A* (9p21.3, 60%). Among these events, *MYC* had the highest rate of high‐level amplification (18.2%), whereas *CDKN2A* showed the most frequent deep deletion (14.2%). We also noted differences in the frequencies of gene‐level CNAs between osteosarcoma and other sarcoma subtypes (Figure S2D); however, the predominance of osteosarcoma in our cohort introduces tumor‐type bias that limits cross‐subtype comparability.

Structural variants (SVs) were analyzed using WGS data, predominantly from osteosarcoma PDXs. WGS of 73 unique PDX models revealed chromothripsis in 93.2% of cases (Figure S3A,B), most often involving chromosomes 1, 2, 6, 8, and 17 (Figure S3C). Among tumor suppressors, *TP53* was most frequently disrupted by SVs (Figure 2D; Table S5), with disruptions in 39 of 73 tumors (53%) and breakpoints predominantly in intron 1 (92.3%). A recent study described loss–translocation–amplification (LTA) chromothripsis in ∼half of high‐grade osteosarcomas, where a single 17p double‐strand break—often disrupting *TP53*—triggers the formation of unstable derivative chromosomes and oncogene amplification [34]. In our PDX cohort, manual review of chromosome 17 identified LTA chromothripsis in 47.1% of osteosarcoma PDXs (Figure S3D–F and Table S8). None of the five non‐osteosarcoma PDXs with WGS data showed *TP53* SV disruptions and LTA chromothripsis. Additionally, we observed recurrent SVs involving tumor suppressor genes, including *RB1* (26%), *ATRX* (18%), *ZFHX3* (16%), *NF2* (16%), *RAD51B* (16%), and *ERBB4* (15%).

Finally, we analyzed gene fusions using RNA‐seq data and identified 2,049 fusions across 143 PDX models (median 12 per model; Figure S4A). Consistent with a recent pediatric PDX study (28), osteosarcoma showed a higher fusion burden than other sarcoma subtypes (Wilcoxon P = 0.044; Figure S4B), but no recurrent oncogenic driver fusions were observed. In contrast, recurrent canonical driver fusions were detected in fusion‐driven sarcomas, including *SS18–SSX1/2* in synovial sarcoma and *EWSR1–FLI1* in Ewing sarcoma (Table S6). Notably, in several cases, fusion results were discordant with the original histopathologic diagnosis, prompting diagnostic reclassification (Table S7). For instance, in SGH‐X‐122, FISH suggested an *EWSR1* rearrangement in both the PDX and the matched patient tumor, but the sample lacked a canonical *EWSR1–FLI1* fusion (Figure S4C); the case was reclassified as osteosarcoma, supported by the presence of a complex genome with features of chromothripsis (Figure S4D,E). In contrast, SGH‐X‐116 was reclassified from osteosarcoma to Ewing sarcoma based on an *EWSR1–FLI1* fusion, which was confirmed by WGS (Figure S4F).

### 2.4 ecDNA‐Driven High‐Level Oncogene Amplification in Osteosarcoma

ecDNA is a major driver of oncogene amplification across human cancers [35], with chromothripsis a key mechanism of formation [36, 37, 38]. Using the short‐read ecDNA detection pipeline AmpliconSuite [39], we identified ecDNA in 44 of 73 unique PDXs (60.3%; Figure 3A; Table S8). Notably, ecDNA was detected exclusively in osteosarcoma PDXs (44/68, 64.7%), with a frequency comparable to that reported in a recent pediatric PDX cohort (68%) [40], and no ecDNA was identified in Ewing sarcoma or chondrosarcoma PDXs. Recurrent osteosarcoma driver oncogenes such as *MYC*, *KIT*, and *CDK4* were most frequently amplified on ecDNA (Figure 3B; Table S9), with significantly higher copy numbers compared to chromosomal amplification (ChrAmp) (Figure 3C).

**FIGURE 3:**
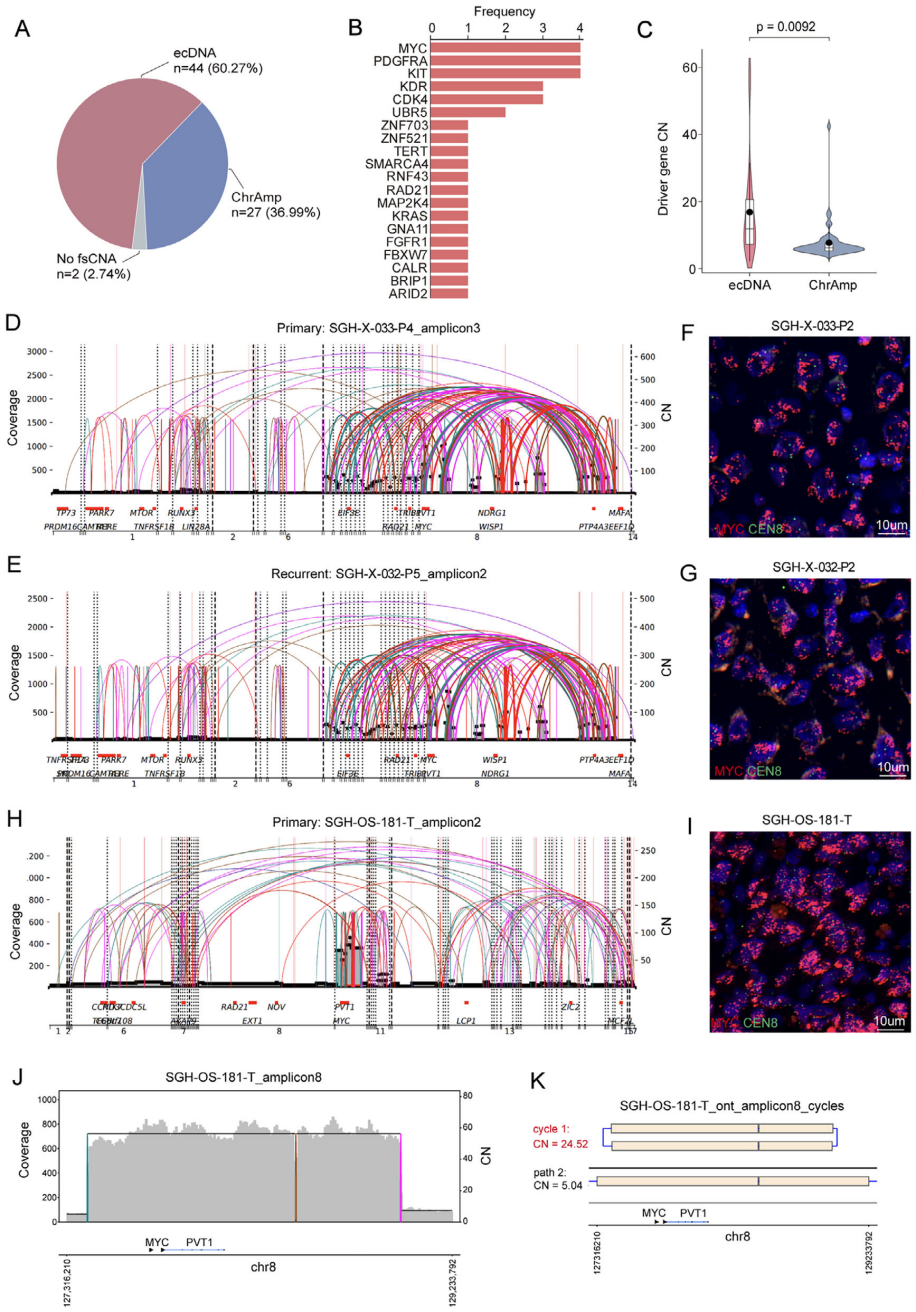
ecDNA is Frequent in Osteosarcoma and Drives High‐Level Oncogene Amplification. (A) Distribution of amplicon status among WGS‐sequenced PDX models, classified as ecDNA, chromosomal amplification (ChrAmp; encompassing BFB, complex non‐cyclic, and linear amplicons as reported by the AmpliconSuite pipeline), or no focal somatic copy number amplification (fsCNA); models harboring both ecDNA and ChrAmp were counted in the ecDNA category. (B) Recurrently amplified driver genes on ecDNA. (C) Copy number of the most highly amplified oncogene for each focal amplification, compared between ecDNA and ChrAmp amplicons; the *p*‐value was calculated using a two‐sided Wilcoxon rank‐sum test. (D, E) SV views of *MYC*‐bearing ecDNA amplicons reconstructed with the AmpliconSuite pipeline in a primary osteosarcoma PDX (D, SGH‐X‐033‐P4) and a recurrent PDX from the same patient (E, SGH‐X‐032‐P5). (F, G) Interphase *MYC/CEN8* FISH images from the corresponding PDXs (SGH‐X‐033‐P2 and SGH‐X‐032‐P2), using dual‐color probes targeting *MYC* (red) and the chromosome 8 centromere *CEN8* (green). Scale bar, 10 µm. (H) SV view of a *MYC*‐bearing ecDNA amplicon in a primary osteosarcoma patient sample (SGH‐OS‐181‐T). (I) Interphase *MYC/CEN8* FISH image from SGH‐OS‐181‐T, using dual‐color probes targeting *MYC* (red) and *CEN8* (green). Scale bar, 10 µm. (J) SV view of the *MYC*‐bearing ecDNA amplicon in SGH‐OS‐181‐T, reconstructed with CoRAL from long‐read sequencing data. (K) CoRAL‐derived reconstruction of the circular *MYC* ecDNA in SGH‐OS‐181‐T, with inferred copy numbers.

Within the cohort, AmpliconSuite identified a highly complex, multichromosomal amplicon in SGH‐X‐111, with *MYC* and *CDK4* amplified on distinct ecDNA species. The amplicon architecture in the PDX closely matched that of the patient tumor (Figure S5A,B). Interphase fluorescence in situ hybridization (FISH) showed numerous multifocal punctate signals (Figure S5C) consistent with ecDNA amplification patterns, whereas metaphase FISH localized these signals to chromosomal sites, predominantly as ectopic intrachromosomal homogeneously staining regions (HSRs) on non‐native chromosomes (Figure S5D). In patient SGH‐124, three PDXs were established from one recurrent and two metastatic samples, all harboring *MYC* amplification, and AmpliconSuite revealed distinct amplicon statuses and architectures, with *MYC* ecDNA in the recurrent PDX (SGH‐X‐142) and ChrAmp in both metastatic PDXs (Figure S5E–G). Interphase FISH confirmed high‐level *MYC* amplification, while metaphase FISH revealed ectopic HSRs in SGH‐X‐142 (Figure S5H). Prior studies suggest that ecDNA and HSR can interconvert [36, 41, 42, 43, 44, 45]; because current bioinformatic methods cannot reliably distinguish circular ecDNA from ecDNA‐derived integrations, we propose that ecDNA reintegration likely contributes to the high‐level ectopic HSRs observed in these two PDX models. Giemsa–Trypsin (GTG)–banded karyotyping of these two PDXs revealed complex tetraploid karyotypes with extensive chromosomal rearrangements and multiple marker chromosomes that were unidentifiable by G‐banding (Figure S5I,J).

In another matched primary tumor and PDX sample (SGH‐027‐D and SGH‐X‐033), whole exome sequencing (WES) showed high‐level *MYC* amplification (Figure S6A). The patient's tumor was highly aggressive, with rapid recurrence 4 months after surgery and death ∼1 year after diagnosis. To delineate the amplicon architecture, we performed short‐read WGS on this pair and on the PDX (SGH‐X‐032) derived from the recurrent tumor. AmpliconSuite confirmed high‐level *MYC* ecDNA in all three samples, with higher *MYC* copy number in the PDXs than in the patient tumor, likely due to the patient sample's low tumor purity (17%) (Figure 3D,E; Figure S6B). Notably, the *MYC* ecDNA amplicon was highly conserved between the primary and recurrent PDXs, indicating stability of this amplicon across the disease course. Interestingly, we also detected a *CCNE1* ecDNA amplicon in the primary PDX (SGH‐X‐033) that was absent from the recurrent PDX (SGH‐X‐032) (Figure S6C). To validate the presence and structure of the ecDNA, we performed interphase FISH and Oxford Nanopore Technologies (ONT) long‐read WGS on both the primary and recurrent PDX samples. Interphase FISH confirmed high‐level *CCNE1* amplification in the primary PDX (SGH‐X‐033), with marked cell‐to‐cell copy number heterogeneity, but no amplification was observed in the recurrent PDX (SGH‐X‐032), (Figure S6D). In addition, long‐read WGS reconstructed a *CCNE1* ecDNA amplicon in SGH‐X‐033, consistent with the short‐read WGS results (Figure S6E). Interphase FISH revealed high‐level MYC ecDNA amplification in both PDXs (Figure 3F–G), and long‐read WGS reconstructed a complex amplicon containing a circular MYC ecDNA in both PDXs (Figure S6F,G). In addition, we included another patient (SGH‐OS‐181) with an aggressive clinical course, presenting with pulmonary metastases at diagnosis and dying ∼1.3 years later. In the primary tumor (SGH‐OS‐181‐T), AmpliconSuite and interphase FISH demonstrated high‐level *MYC* ecDNA amplification (Figure 3H,I), and long‐read WGS reconstructed a circular *MYC* ecDNA (Figure 3J,K). Collectively, these findings indicate that ecDNA frequently drives high‐level oncogene amplification in osteosarcoma and that *MYC* ecDNA may underlie particularly aggressive disease trajectories.

### 2.5 Divergent Inheritance of ChrAmp and ecDNA during PDX Engraftment

Unlike ChrAmp, ecDNA lacks centromeres and segregates unevenly during mitosis, driving high‐level oncogene amplification and promoting tumor evolution and heterogeneity [46]. Longitudinal pan‐cancer analyses show that ecDNA is more frequently retained over the disease course than ChrAmp [47]. We investigated differences in the inheritance of ecDNA and ChrAmp during engraftment by analyzing 24 matched patient and PDX WGS pairs, restricting samples to patient tumors with purity ≥30% to limit purity‐related bias. Amplicon status was highly concordant between patients and matched PDXs. Two PDXs contained ecDNA that was not detected in the corresponding patient tumors, and one PDX harbored a ChrAmp not detected in the patient (Figure 4A). However, manual review in each case revealed low copy numbers in both samples, indicating low‐confidence calls rather than *de novo* emergence.

**FIGURE 4:**
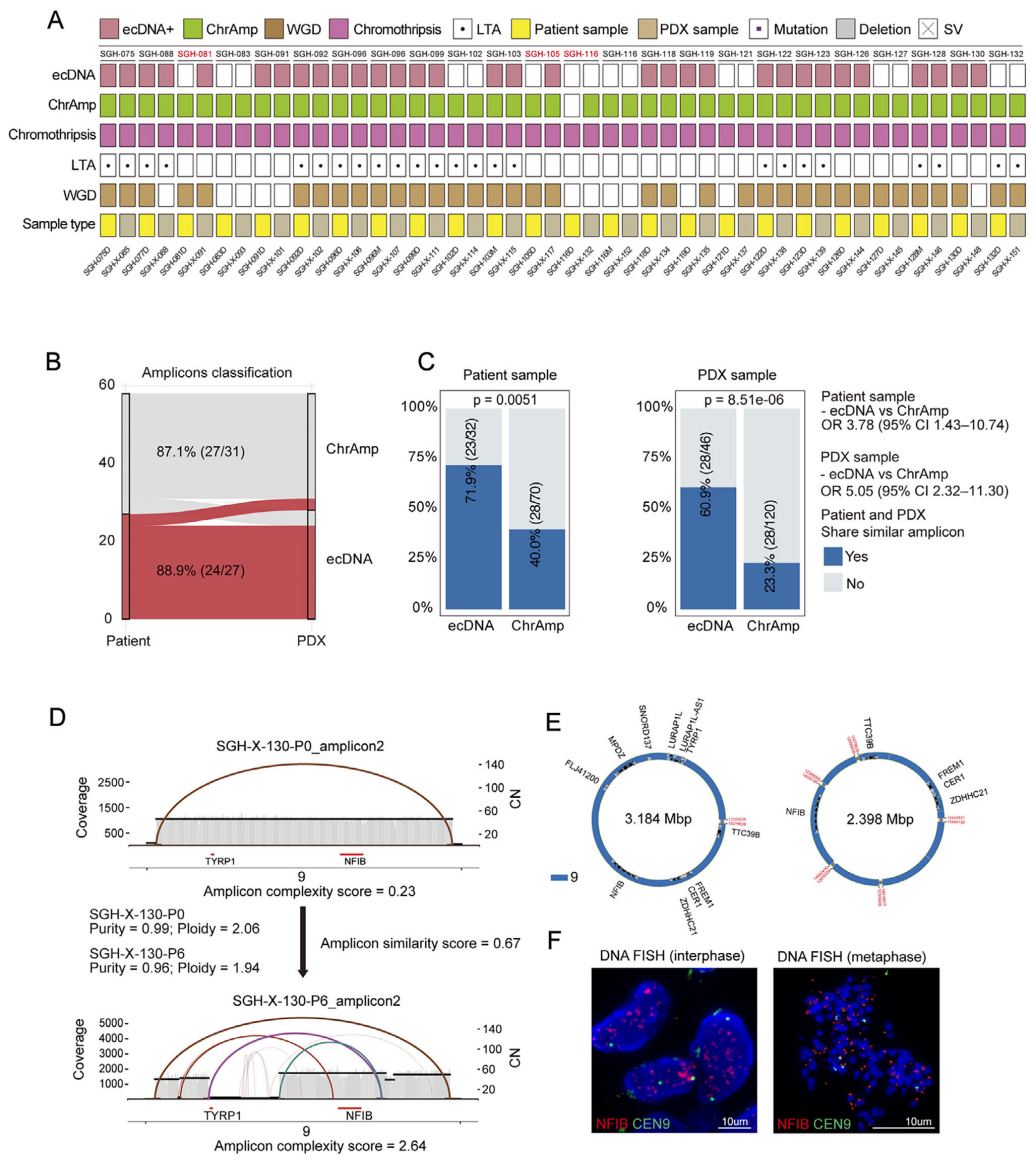
ecDNA Is Preferentially Retained Relative to ChrAmp During PDX Engraftment. (A) Heatmap of amplicon status and genomic features in 24 high‐purity osteosarcoma patient–PDX pairs. Columns show samples grouped by patient–PDX pairs. Sample IDs in red mark discordant amplicon status between the patient and PDX. (B) Sankey plot showing changes in amplicon status between patient tumors and PDXs for 58 paired amplicons with significant similarity; percentages indicate the fraction of amplicons that retain their original class. (C) Fraction of ecDNA and ChrAmp amplicons shared between the patient tumors and matched PDXs (patient‐indexed, left; PDX‐indexed, right); numbers in parentheses show shared/total amplicons, and *p*‐values were calculated using Fisher's exact test. (D) SV views of *NFIB*‐bearing ecDNA amplicons in SGH‐X‐130 at early (P0) and late PDX passage (P6). (E) Circular structures of *NFIB*‐bearing ecDNA in SGH‐X‐130 at early (P0, left) and late passage (P6, right), reconstructed with CycleViz; SV breakpoints (genomic coordinates) are shown in red. (F) Interphase and metaphase *NFIB/CEN9* DNA FISH images from SGH‐X‐130 using dual‐color probes targeting *NFIB* (red) and *CEN9* (green). Scale bar, 10 µm.

We next computed similarity scores from shared breakpoints and genomic content and identified 58 matched amplicon pairs (Table S10). Four pairs (6.9%) were ChrAmp in the patients but ecDNA in the PDXs, whereas three pairs (5.2%) showed the reverse pattern (Figure 4B). To exclude potential bias introduced by downsampling, we reanalyzed the data using full‐coverage BAM files, which reduced this to three persistent ChrAmp‐to‐ecDNA conversions. Among these, the first two amplicon pairs showed highly similar architectures between the patient tumor and matched PDX but low copy numbers, consistent with low‐confidence calls (Figure S7A,B). In contrast, the remaining pair showed significantly increased copy numbers relative to the matched patient (Figure S7C). An ecDNA amplicon was also identified in the patient tumor, but was present at substantially higher copy number in the matched PDX (Figure S7D). These findings are consistent with previous studies, which indicate that ecDNA‐bearing clones may gain a fitness advantage during engraftment, resulting in substantially higher oncogene copy numbers in PDXs than in the matched patient tumors [48, 49, 50].

ecDNA was shared between patient tumors and matched PDXs significantly more often than ChrAmp (Figure 4C), indicating preferential and more stable retention of ecDNA over ChrAmp during engraftment. There were no significant differences in amplicon size, copy number, or structural complexity when comparing paired amplicons from each PDX and its matched tumor (Figure S8A). Interestingly, we observed structural evolution of ecDNA across long‐term passaging in one PDX model (Figure 4D). In SGH‐X‐130, AmpliconSuite identified an ecDNA on chromosome 9 containing the *NFIB* oncogene. The P0 amplicon was ∼3.18 Mb with a low amplicon complexity score of 0.23. At P6, the ecDNA had shortened to ∼2.40 Mb but acquired additional junctions, and the amplicon complexity score increased to 2.64 (Figure 4E). GTG‐banded karyotyping demonstrated highly complex near‐triploid karyotypes with extensive chromosomal rearrangements and numerous double minutes, a cytogenetic hallmark of ecDNA (Figure S8B). FISH confirmed the *NFIB* ecDNA nature of this amplicon (Figure 4F), and long‐read sequencing reconstructed a circular amplicon containing *NFIB* (Figure S8C,D). Collectively, these data indicate that ecDNA is preferentially retained over ChrAmp during PDX engraftment. In addition, ecDNA can undergo structural evolution during long‐term passaging while preserving its core oncogene architecture.

### 2.6 Histopathological and Molecular Fidelity of PDXs Compared to Patient Tumors

To assess histopathological fidelity, we compared matched patient–PDX pairs across eight sarcoma subtypes (Figure 5A). Hematoxylin and eosin (H&E)‐stained slides showed close morphologic concordance between PDXs and the corresponding patient tumors. Immunohistochemistry (IHC) showed marker expression consistent with the patients’ tumors, including SATB2 in osteosarcoma, H3G34W in giant cell tumor of bone (GCTB), and H3K36M in chondroblastoma. In parallel, FISH confirmed hallmark genomic alterations in both PDX and patient samples, including *SS18* rearrangement in synovial sarcoma, *EWSR1* rearrangement in Ewing sarcoma, and *MDM2* amplification in liposarcoma. We further examined three osteosarcoma subtypes and confirmed that PDXs recapitulate the histologic features and key immunohistochemical markers of the corresponding patient tumors (Figure 5B), including similar bone formation in osteoblastic tumors, cartilage‐like matrix in chondroblastic tumors, and fibroblastic morphology in fibroblastic tumors. PDXs had significantly higher tumor purity than patient tumors, whereas ploidy showed no significant changes (Figure 5C). Enrichment analysis of RNA‐seq data from paired PDX and patient tumors showed MYC pathway activation and significant suppression of immune‐related pathways in PDX samples (Figure S9A). Immune and stromal signature scores were consistently and markedly reduced in PDXs relative to their matched patient tumors (Figure 5D). These results align with previous studies and indicate that engraftment depletes stromal and immune components while enriching malignant cells within the xenograft microenvironment [51, 52, 53].

**FIGURE 5:**
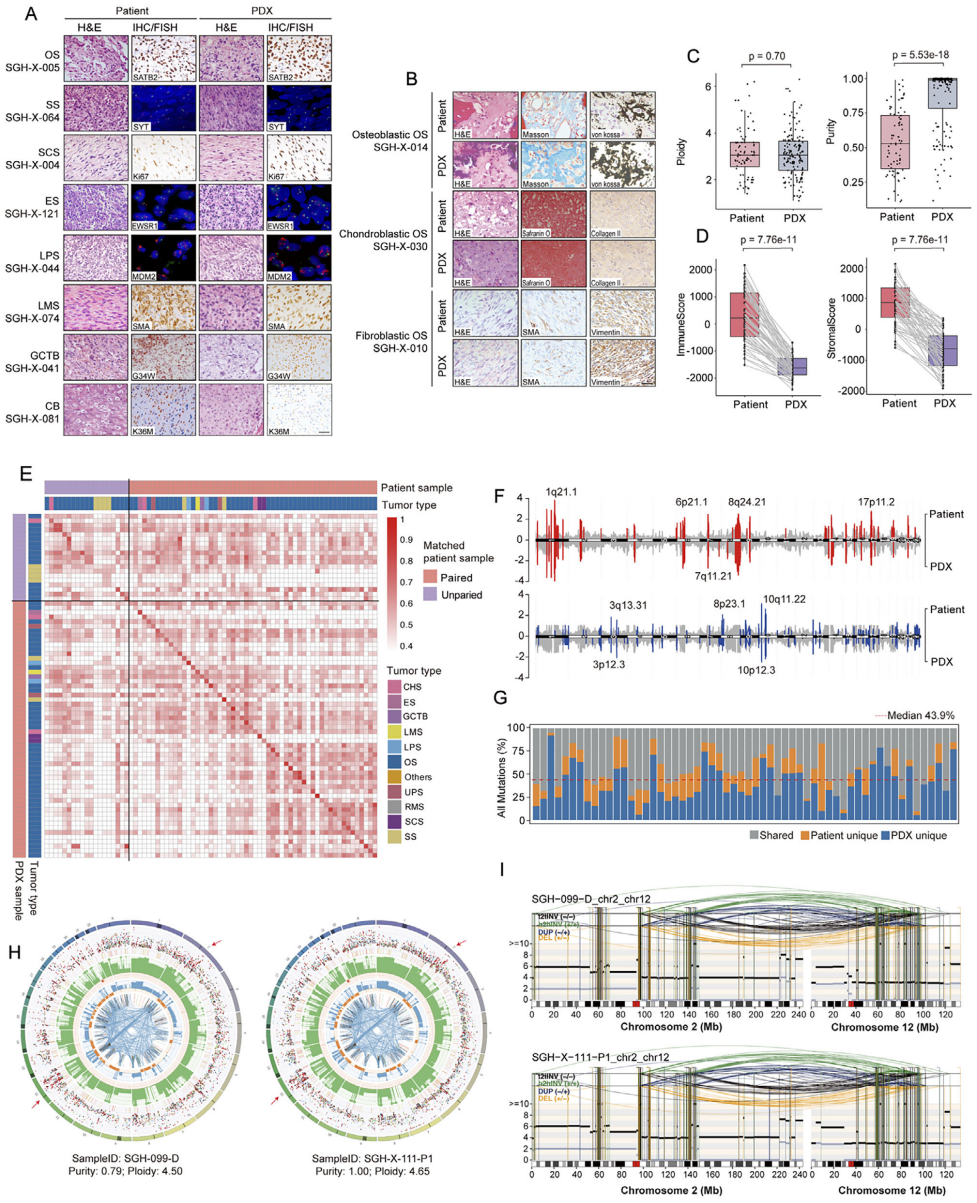
PDXs Recapitulate the Histologic and Molecular Features of Matched Patient Tumors. (A) Representative H&E and IHC/FISH images from patient tumors and matched PDXs across multiple sarcoma histotypes. Scale bars, 50 µm for H&E/IHC and 10 µm for FISH. (B) Representative H&E and IHC images of three osteosarcoma subtypes (osteoblastic, chondroblastic, and fibroblastic) in patient tumors and matched PDXs. Scale bar, 50 µm. (C) Ploidy (left) and purity (right) estimated from WES/WGS data in 76 patient tumors and 148 PDX samples; *p*‐values were calculated using the Wilcoxon rank‐sum test. (D) ImmuneScore (left) and StromalScore (right) estimated from RNA‐seq for 56 paired patient–PDX samples; *p*‐values were calculated using the paired Wilcoxon signed‐rank test. (E) Spearman correlation heatmap of RNA‐seq profiles for all patient tumors (columns) and PDXs (rows), computed from the top 2,000 most variable protein‐coding genes. Annotation bars indicate tumor type and pairing status (paired, PDX derived from the same tumor sample that was RNA‐sequenced in the patient; unpaired, PDX derived from a different tumor sample from the same patient). (F) Genome‐wide comparison of CNAs between WGS‐profiled osteosarcoma patient tumors and PDX models, with CNA gains (top) and losses (bottom) plotted separately. (G) Concordance of somatic mutations between matched patient tumors and PDXs. Stacked bars show shared, patient‐unique, and PDX‐unique mutations; the dashed line indicates the median fraction of shared mutations. (H) Circos plots showing the landscape of somatic alterations from WGS for an osteosarcoma patient–PDX pair (patient, left; PDX, right); From the outermost to the innermost track, the plots show chromosome ideograms, somatic SNVs/indels, total copy number, minor‐allele copy number, and structural variant links; red arrows highlight prominent translocations between chromosomes 2 and 12. (I) Somatic SV and copy number profiles of chromosomes 2 and 12 for the matched patient–PDX pair (patient, top; PDX, bottom), visualized using ReConPlot. DEL, deletion‐like rearrangement; DUP, duplication‐like rearrangement; h2hINV, head‐to‐head inversion; t2tINV, tail‐to‐tail inversion.

We next evaluated the molecular concordance between PDXs and patient tumors across distinct omics levels. At the transcriptomic level, we observed high correlations between PDXs and patient tumors (median Spearman correlation: 0.74; IQR: 0.65–0.80; Figure 5E), with high consistency across different passages (Figure S9B). At the CNA level, WGS data from osteosarcoma revealed highly concordant amplification and deletion peaks between PDXs and patient tumors (Figure 5F). To minimize the impact of tumor purity on CNA analysis, we selected 58 matched patient–PDX pairs with tumor purity ≥30% for gene‐level CNA analysis, and the results demonstrated a strong correlation (Spearman correlation median: 0.76; IQR: 0.57–0.83, Figure S9C). For somatic mutations, PDXs carried more detectable variants than their matched tumors (Figure S9D), consistent with prior reports and likely reflecting the higher tumor purity of PDX samples [25]. Notably, the median mutational similarity between PDXs and patient tumors in our cohort was 43.9% (Figure 5G), increasing to 47.5% when restricted to driver genes (Figure S9E), but remaining lower than the previously reported 52% in childhood solid cancers [27] and 75% in adult cancers [54].

Using WGBS data from osteosarcoma cases, we observed strong concordance in genome‐wide DNA methylation profiles between PDXs and patient tumors (median Spearman 0.82, IQR 0.64–0.86; Figure S9F), indicating that epigenomic features are also largely preserved during engraftment. Finally, we compared the number of SVs between PDX models and their matched patient tumors. Overall, SV burden was higher in PDX models compared with matched patient tumors, potentially reflecting their higher tumor purity (Figure S9G). In addition, in our cohort, patient sample SGH‐099‐D exhibited highly complex genomic rearrangements and the highest SV burden in patient tumors, and its matched PDX (SGH‐X‐111‐P1) preserved these alterations (Figure 5H), including dense translocations between chromosomes 2 and 12 (Figure 5I). Collectively, our results demonstrate that PDX models largely recapitulate the histological and molecular features of their matched patient tumors.

### 2.7 Clonal Evolution Dynamics During PDX Engraftment

To assess clonal dynamics during PDX engraftment, we performed clonal analysis of somatic mutations from 47 paired patient–PDX samples with matched normal DNA and tumor purity ≥30%, predominantly from osteosarcoma cases. Across these patient–PDX pairs, 1,106 mutations were retained in the PDX models, including 960 clonal and 146 subclonal mutations (Figure S10A). Among the retained mutations, 83.2% (799/960) of clonal mutations in the patient tumors remained clonal in the PDXs, whereas 71.2% (104/146) of subclonal mutations became clonal in the matched PDXs. Of the 799 retained clonal mutations, 91.1% (728/799) arose from osteosarcoma cases (n = 40; Figure 6A) and involved well‐established cancer driver genes in osteosarcoma, such as *TP53* and *RB1* (Figure 6B). The remaining 8.9% (71/799) were derived from other sarcomas and involved only two driver genes, *LRP1B* and *CREBBP* (*n* = 7; Figure 6C).

**FIGURE 6:**
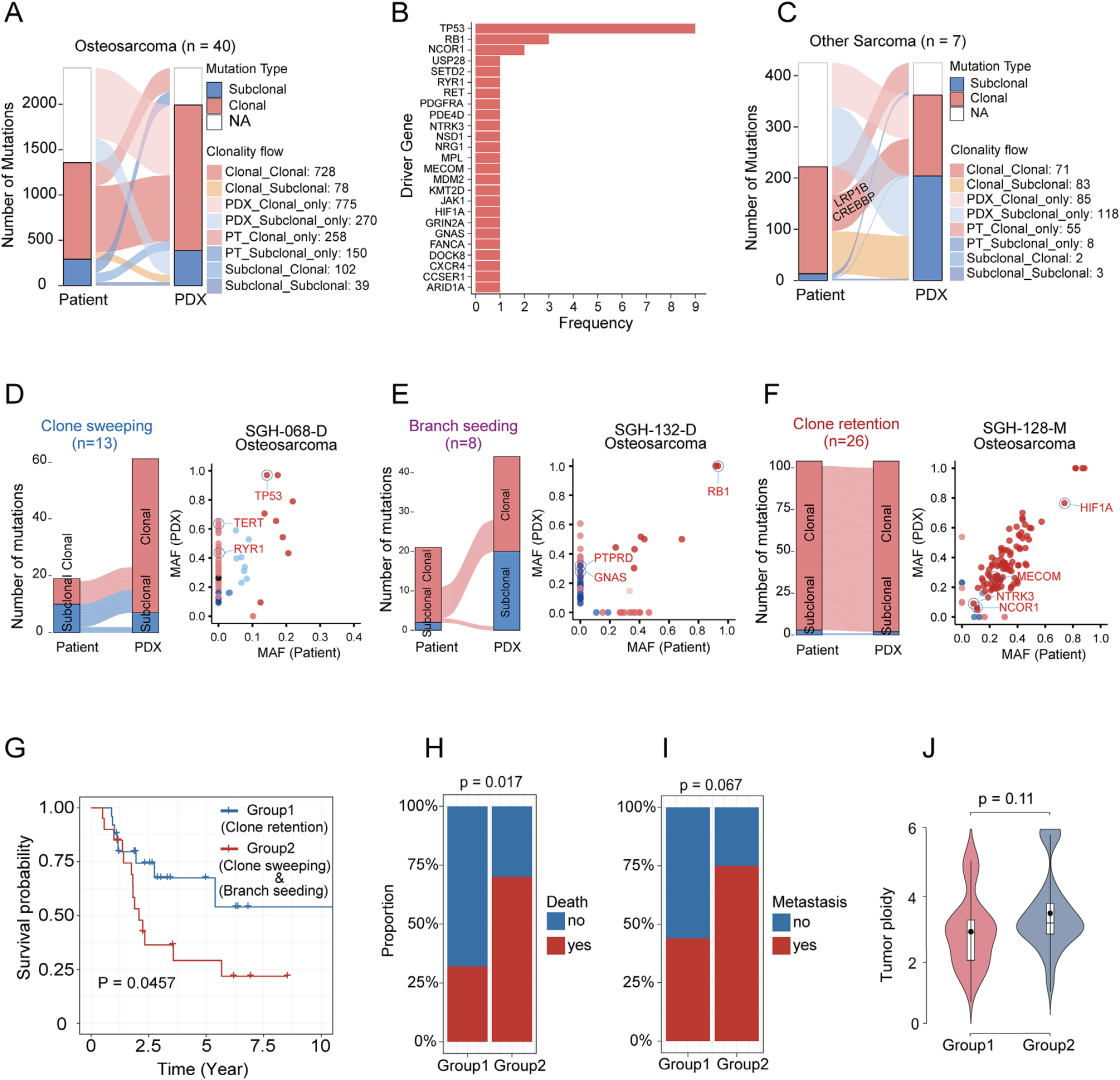
Distinct Clonal Evolution Patterns During PDX Engraftment. (A) Alluvial diagram showing mutation clonality flow from patient tumor to PDX for 40 paired osteosarcoma samples; clonal mutations are in red and subclonal mutations in blue, and band width reflects the number of mutations. (B) Top driver genes among clonal mutations retained in osteosarcoma PDX models. (C) Alluvial diagram of mutation clonality flow from patient tumor to PDX for seven paired “other sarcoma” samples. (D–F) Representative examples of clone sweeping (D), branch seeding (E), and clone retention (F). Left, alluvial diagrams of clonality flow; right, scatter plots of tumor mutant allele fraction (MAF) in patient (x‐axis) versus PDX (y‐axis), with driver genes labeled in red. (G) Kaplan–Meier curves comparing overall survival between Group 1 (clone retention) and Group 2 (clone sweeping and branch seeding); the *p*‐value was calculated using the log‐rank test. (H–J) Mortality (H), metastatic incidence (I), and tumor ploidy (J) were compared between Group 1 and Group 2; *p*‐values were calculated by Fisher's exact test and Wilcoxon rank‐sum test (J).

We next classified paired patient–PDX samples into three evolutionary patterns using a previously described framework based on changes in mutation clonality between patient tumors and matched PDXs [27]. The first pattern, clone sweeping (*n* = 13), was characterized by subclonal mutations in the patient tumor becoming clonal in the matched PDX, consistent with the expansion of pre‐existing minor subclones (Figure 6D; Figure S10B). The second pattern, branch seeding (*n* = 8), was characterized by loss of most clonal mutations present in the patient tumor, with only a limited set of early clonal mutations retained in the PDX, suggesting engraftment from a minor branch of the tumor phylogeny (Figure 6E; Figure S10C). The third pattern, clone retention (n = 26), was the most common and was characterized by preservation of clonal mutations from the patient tumor in the matched PDX, together with a broadly similar clonal composition (Figure 6F; Figure S10D). Interestingly, patients with the clone‐retention pattern had more favorable overall survival than those with clone‐sweeping or branch‐seeding patterns (log‐rank *p* = 0.0457; Figure 6G). Consistently, patients with clone‐sweeping or branch‐seeding patterns exhibited higher mortality compared with those with the clone‐retention pattern (70.0% vs 32.0%; Fisher's exact test *p* = 0.017; Figure 6H), a higher incidence of metastasis (75.0% vs 44.0%; Fisher's exact test *p* = 0.067; Figure 6I), and a trend toward increased tumor ploidy (median 3.15 vs 2.82; Wilcoxon rank‐sum test *p* = 0.11; Figure 6J). These results suggest an association between clone‐sweeping or branch‐seeding patterns and greater genomic instability and intratumoral heterogeneity, consistent with previous reports [25, 27, 54, 55].

### 2.8 Therapeutic Translational Potential of the SGH Sarcoma PDX Cohort

We used the FDA‐recognized OncoKB precision oncology knowledge base (https://www.oncokb.org) to evaluate the therapeutic actionability of genomic alterations in our PDX cohort [56, 57]. Alterations were assigned to four evidence levels, from Level 1 (FDA‐approved biomarker in the matched indication) to Level 4 (supported by preclinical data). Among 153 PDX models with available sequencing data, 80 (52.3%) carried at least one potentially actionable alteration (Figure 7A). The majority of alterations were Level 3 or 4, indicating investigational relevance, and only ivosidenib for IDH1‐mutant chondrosarcoma was classified as Level 2 (Figure 7B) [58, 59].

**FIGURE 7:**
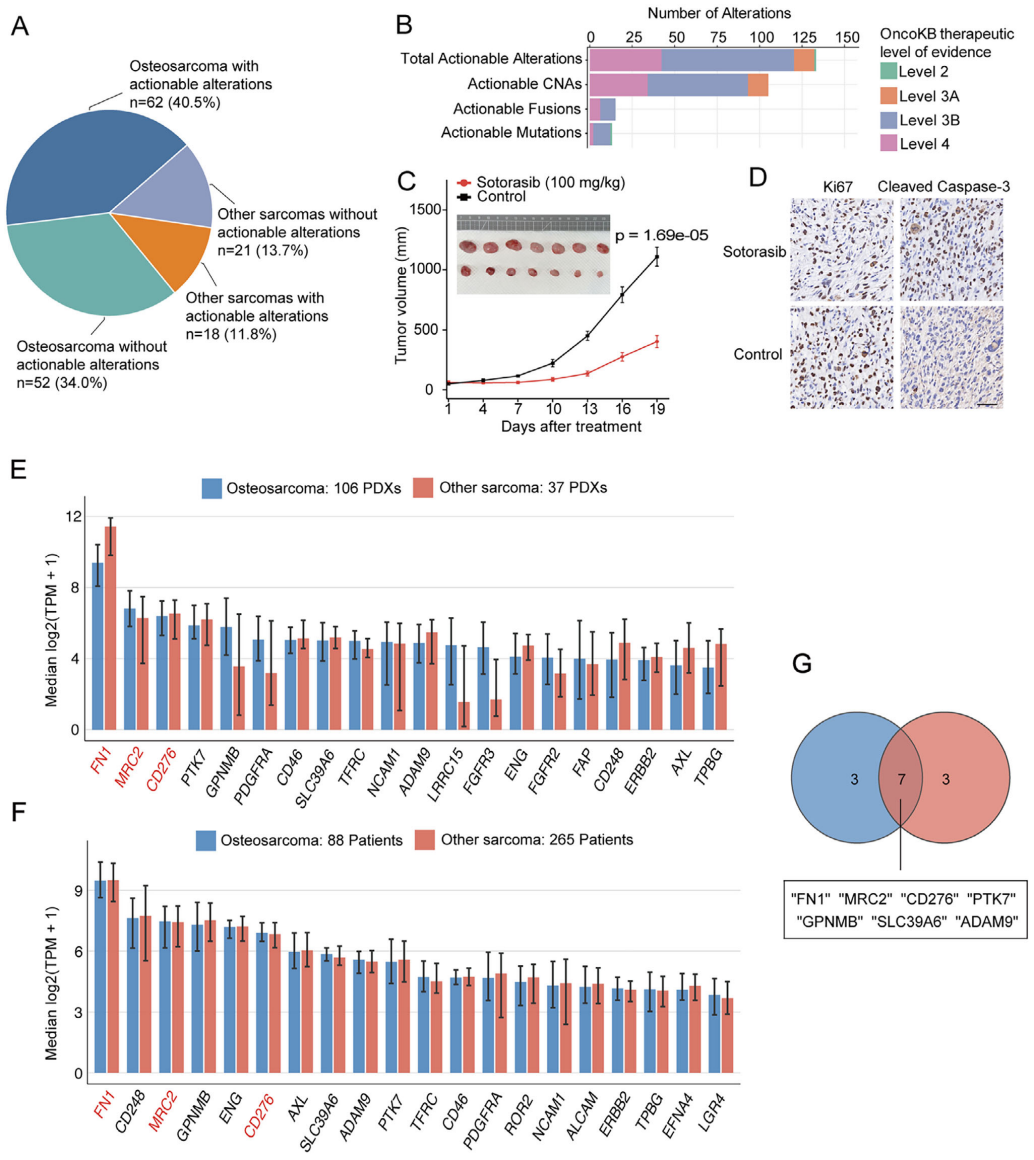
Therapeutic Translational Potential of the SGH Sarcoma PDX Cohort. (A) Distribution of potentially actionable genomic alterations in 153 unique PDX models, stratified by osteosarcoma versus other sarcoma and by presence or absence of actionable events. (B) OncoKB therapeutic evidence levels for potentially actionable alterations, shown separately for all events, actionable CNAs, fusions, and mutations. (C) Tumor growth curves for a KRAS p.G12C–mutant PDX model (SGH‐X‐089‐P1) treated with vehicle or sotorasib (100 mg/kg; *n* = 7 per group; data are mean ± SEM); the *p‐*value was calculated using the two‐tailed unpaired Student's *t*‐test. (D) Representative IHC staining for Ki‐67, and cleaved caspase‐3 in tumors from sotorasib‐treated versus vehicle‐treated mice. (E) Expression of the top 20 of 62 ADC target genes in osteosarcoma (*n* = 106) and other sarcoma (*n* = 37) PDX models, with genes ranked by overall expression across all PDXs; error bars indicate the interquartile range. (F) Expression of the top 20 of 62 ADC target genes in the TARGET osteosarcoma cohort (*n* = 88) and the TCGA sarcoma cohort (*n* = 265), with genes ranked by overall expression across all patients; error bars indicate the interquartile range. (G) Venn diagram showing the overlap between the top 10 highly expressed ADC targets in the PDX cohort and the patient cohort; genes in the intersection represent shared ADC targets.

In our PDX cohort, the most frequent potentially actionable alterations were deletions of *CDKN2A*, followed by *MTAP* and *PTEN*, whereas potentially actionable amplifications were rare and limited to *CCNE1*, *MET*, and *MDM2* (Tables S11 and S12). Recurrent actionable mutations or fusions were uncommon, except for the *EWSR1–FLI1* fusion in Ewing sarcoma. Notably, we identified a *KRAS* p.G12C hotspot mutation in one osteosarcoma PDX model (SGH‐X‐089‐P1). Sotorasib, a selective *KRAS* G12C inhibitor approved for non‐small‐cell lung cancer [60], has demonstrated activity in multiple tumor types carrying this mutation [61, 62]. In this model, sotorasib treatment significantly inhibited tumor growth (Figure 7C). Immunohistochemistry further demonstrated reduced Ki‐67 expression and increased cleaved caspase‐3 levels in sotorasib‐treated tumors (Figure 7D), supporting the utility of our sarcoma PDX cohort for preclinical evaluation of genome‐matched targeted therapies.

Finally, we analyzed the expression of 62 manually curated antibody–drug conjugate (ADC) targets from a previous study [63], and identified several highly expressed candidate targets in our PDX cohort, including CD276 (B7‐H3) and MRC2 (Figure 7E), both previously reported to be highly expressed in diverse sarcoma histotypes [64, 65, 66]. We then evaluated these candidates in external validation cohorts using TARGET osteosarcoma and TCGA sarcoma RNA‐seq data, which confirmed their high expression in patient tumors (Figure 7F) and showed substantial overlap in top‐ranked ADC targets between the PDX and patient datasets (Figure 7G). Notably, in our analysis, FN1, which encodes fibronectin, a ubiquitously expressed high‐molecular‐weight extracellular matrix glycoprotein [67, 68], emerged as the most highly expressed ADC candidate in both cohorts.

## 3 Discussion

In this study, we established the SGH sarcoma PDX biobank, a multi‐omics resource of 155 models from 132 patients across 11 sarcoma histotypes, with osteosarcoma predominating. Each model has been profiled at the genomic and transcriptomic levels with detailed clinical annotation. Importantly, we found that successful PDX engraftment enriches for biologically aggressive disease and represents a clinically high‐risk sarcoma subset, underscoring the value of this resource for sarcoma research. Although the present work is primarily computational, these PDXs have been used in our previous studies [19, 69, 70, 71, 72]. For example, in osteosarcoma, we identified subtype‐specific vulnerabilities, with the HRD‐like subtype responding to PARP inhibition and the MYC‐driven subtype showing sensitivity to the Aurora kinase inhibitor tinengotinib (TT‐00420) [19, 73]. Using GCTB PDX models, we showed that HDAC inhibition with ITF‐2357 significantly suppresses GCTB progression [69]. In addition, organoids can be generated from established PDX tumors, and using such models, our recent work showed that a PRMT5^MTA^ inhibitor enhances immunotherapy response in chromosome 9p21.3‐deleted osteosarcoma [74]. These studies highlight the utility of our PDX platform for translational research, and here we provide this living multi‐omic PDX resource to the scientific community to further advance sarcoma genomics and the preclinical evaluation of cancer therapeutics.

A key finding of this study was that ecDNA was frequent in osteosarcoma PDX models, with recurrent driver oncogenes most often amplified as ecDNA. Our previous multi‐omics study [19], together with other reports [30, 31], demonstrated that *MYC* amplification is a defining feature of high‐risk osteosarcoma. By integrating short‐read and long‐read WGS with FISH, we found that ecDNA frequently underlies high‐level *MYC* amplification in osteosarcoma. Interestingly, in two PDX models (SGH‐X‐111 and SGH‐X‐142), ecDNA was identified by AmpliconSuite; however, metaphase FISH revealed high‐level ectopic amplification. Prior studies have described interconversion between ecDNA and HSR, suggesting a dynamic relationship between these amplification states [36, 41, 42, 43, 44, 45]. Several studies suggest that ecDNA is not just a by‐product of genomic instability but can further remodel the cancer genome [75, 76]. A neuroblastoma study showed that *MYCN* ecDNA can drive oncogenic genome remodeling through chimeric circularization and subsequent reintegration into chromosomal loci [76]. A pan‐cancer study identified an amplification pattern termed “seismic amplification,” characterized by complex rearrangements and discontinuous copy number levels [75]. In this model, chromothripsis generates ecDNA that undergoes repeated circular recombination, and then either persists as double minutes or reintegrates into chromosomes as ectopic ChrAmp [75]. Experimental evidence has shown that ecDNA can reintegrate at chromosomal breakpoints to generate ectopic ChrAmp [36]. These studies provide a plausible explanation for the high‐level ectopic ChrAmp observed in our two karyotypically complex PDX models.

Interestingly, in another paired primary–recurrent PDX series (SGH‐X‐033 and SGH‐X‐032), *MYC* ecDNA was highly conserved, whereas a *CCNE1* ecDNA detected in the primary PDX was absent in the recurrent PDX. Given that our analysis of paired patient–PDX samples did not reveal convincing examples of ecDNA loss during engraftment, this pattern is more consistent with loss of the *CCNE1* ecDNA during tumor progression. A similar phenomenon has been reported in gliomas, where *EGFR* ecDNA is present in primary tumors but absent in the recurrent tumors [77]. Although spatial heterogeneity (e.g., resection of an ecDNA‐containing subclone in the primary tumor) may partly explain this pattern [77], another possibility is that distinct ecDNA species confer different selective advantages during disease progression [35]. However, given the highly complex and heterogeneous genome of osteosarcoma, clarifying the role of ecDNA in genomic remodeling and disease progression will still require larger longitudinal cohorts and orthogonal approaches, including single‐cell sequencing (e.g., scWGS, scATAC‐seq) and 3D genome (Hi‐C) profiling.

Across multiple sarcoma subtypes, our analyses showed that PDX models largely recapitulate their matched patient tumors at both the histopathologic and multi‐omics levels. However, point‐mutation concordance between PDXs and matched patient tumors was only moderate, consistent with previous reports and likely reflecting lower tumor purity, limited sequencing depth, and clonal evolution during engraftment [25, 27]. We identified the three previously described clonal evolution patterns [27], with the clone‐retention pattern associated with better survival and the other two patterns linked to poorer outcomes, likely reflecting greater genomic instability and heterogeneity in the primary tumors [78]. In contrast, PDXs and matched patient tumors showed high concordance at the SV and CNA levels, consistent with the notion that non‐fusion‐driven sarcomas—particularly osteosarcoma—are driven predominantly by SVs and CNAs rather than by point mutations [24, 34].

We also evaluated the translational potential of this PDX resource. Analysis of targetable genomic alterations showed that 52.3% of models carried at least one potentially actionable alteration, and in an osteosarcoma PDX harboring a *KRAS* p.G12C mutation, sotorasib treatment markedly suppressed tumor growth. ADCs have demonstrated remarkable efficacy across diverse malignancies and are reshaping cancer therapy [79]. By analyzing 62 manually curated ADC targets, we found that FN1 was highly expressed across multiple sarcoma subtypes. Notably, its extra domain B splice isoform (EDB‐FN) is largely absent from normal tissues but is upregulated in the tumor microenvironment across multiple solid tumors [80, 81]. Unlike most membrane‐bound ADC targets, FN1, particularly its EDB‐FN isoform, is enriched in the tumor stroma but largely absent from most normal tissues [80, 81]. PYX‐201 is an investigational ADC that targets EDB‐FN in the tumor stroma and has shown favorable tolerability and robust antitumor activity in preclinical PDX models, including multiple sarcoma subtypes [82, 83, 84, 85], suggesting that EDB‐FN may represent a promising ADC target in sarcoma.

Several limitations of this study should be acknowledged. First, our PDX cohort is heavily enriched for osteosarcoma, and some rarer sarcoma subtypes are sparsely represented. Second, approximately half of the PDXs lacked matched germline DNA. Although we applied stringent filtering to reduce contamination by germline variants, somatic variant calls in these models should still be interpreted with caution. Therefore, in preclinical studies evaluating therapies directed at specific genomic alterations in these models, orthogonal validation with higher‐depth targeted sequencing, IHC, or FISH will be required. Meanwhile, although we prioritized candidate ADC targets based on transcriptomic profiling, additional protein‐level validation will be essential before these candidates can be translated into clinical applications. Third, because most WGS data and ecDNA analyses in our study were generated in osteosarcoma, the prevalence of ecDNA in other sarcoma subtypes remains incompletely defined and will require larger, histology‐balanced cohorts to clarify.

## 4 Methods

### 4.1 Patient Sample Collection and PDX Model Establishment

This study was approved by the Institutional Research Ethics Committee of Shanghai General Hospital, and written informed consent was obtained from all participants. Fresh tumor samples obtained from patients undergoing surgical resection or biopsy at Shanghai General Hospital were immediately placed in 15 mL polypropylene tubes containing serum‐free DMEM and transported on wet ice. One portion of these samples was sent to the pathology department for histopathological evaluation, while the remaining portion was transferred to the research laboratory for the establishment of PDX models. Upon arrival, the samples were washed thoroughly, and tumor fragments measuring 3–5 mm in size were subcutaneously implanted into the flanks of 6–8‐week‐old female NSG mice. Recipient mice were monitored for up to 6 months after implantation. Passage 0 (P0) was defined as the first‐generation xenograft directly established from the implanted patient tumor. Successful PDX engraftment was defined as successful tumor establishment followed by successful propagation to at least passage 1 (P1). When tumors reached a volume of 800–1200 mm^3^, mice were euthanized via cervical dislocation. The tumors were aseptically excised and placed in a sterile culture dish, then cut into 1 × 1 × 1 mm^3^ fragments. Part of the tissue was used for serial passaging through subcutaneous flank implantation into new NSG mice, while the remaining portions were processed for biobanking through cryopreservation and formalin‐fixed paraffin embedding (FFPE) for long‐term storage. All procedures related to animal welfare were reviewed and approved by the Institutional Animal Care and Use Committee (IACUC) of Shanghai General Hospital.

### 4.2 DNA Sequencing and Data Preprocessing

The details of the whole‐exome sequencing (WES) have been described previously [19]. Whole‐genome sequencing (WGS) in this study was performed primarily by Shanghai Sequanta Technologies Co., Ltd., with a subset of samples processed by Wuhan Benagen Technology Co., Ltd. For Sequanta, genomic DNA (gDNA) was extracted from ∼10 mg of tissue using the QIAamp DNA Mini Kit (Qiagen) and from 1 mL of peripheral blood using the QIAamp DNA Blood Mini Kit (Qiagen), according to the manufacturers’ instructions. DNA integrity was assessed by agarose gel electrophoresis, and DNA quantity and purity were evaluated using a Qubit 3.0 fluorometer with the dsDNA HS Assay Kit and a NanoDrop 2000 spectrophotometer (Thermo Fisher Scientific). For WGS, 200–300 ng of high‐quality gDNA (OD260/280 = 1.8–2.0) was fragmented to an average size of 150–200 bp using a Covaris LE220 sonicator, and libraries were prepared with the TruSeq Nano DNA HT Sample Prep Kit (Illumina), including end repair, AMPure XP bead purification, A‐tailing, adapter ligation, and PCR amplification. Final libraries were quantified by Qubit, sized on an Agilent BioAnalyzer (Agilent Technologies), and sequenced as 2 × 150 bp paired‐end reads on Illumina NovaSeq 6000 platforms (RRID:SCR_016387), targeting ∼60× coverage for both tumor and matched germline samples.

For samples processed by Wuhan Benagen Technology Co., Ltd., gDNA from animal tissues (∼50 mg) and peripheral blood was extracted using an SDS‐based lysis and organic extraction protocol. Frozen tissues were mechanically homogenized in SDS lysis buffer, and blood cell pellets (prepared by two washes in 1× PBS) were lysed in the same buffer with RNase A, followed by incubation at 50°C for 1 h. After centrifugation, DNA was purified by phenol/chloroform/isoamyl alcohol and chloroform/isoamyl alcohol extraction, precipitated with cold isopropanol, washed with 75% ethanol, air‐dried, and resuspended in QIAGEN Elution Buffer. DNA quantity and purity were assessed by NanoDrop One spectrophotometry, Qubit 3.0 fluorometry, and 1% agarose gel electrophoresis. For WGS library preparation, qualified DNA was fragmented to ∼350 bp using an ultrasonic processor, followed by end repair, A‐tailing, adapter ligation, purification, and PCR amplification to generate ∼350 bp insert libraries. Libraries were quantified by Qubit, evaluated on an Agilent 2100 BioAnalyzer, and sequenced as 2 × 150 bp paired‐end reads on a DNBSEQ‐T7 platform (RRID:SCR_017981), targeting ∼50–60× coverage for tumors and ∼30× coverage for matched germline samples.

Raw sequencing data were processed to trim poor‐quality reads and adapters using Trim Galore (v0.6.10; RRID:SCR_011847) [86]. To remove mouse reads from PDX sequences, the trimmed reads were aligned to both the human hs37d5 and mouse mm10 genomes using BBsplit from BBMap (v37.00; RRID:SCR_016965). Reads with high probability of mapping to the human genome were retained and re‐aligned to the hs37d5 reference genome using BWA‐MEM (v0.7.17; RRID:SCR_010910) [87]. The BAM files were sorted with Samtools (v1.17; RRID:SCR_002105) [88], and duplicate reads were marked using the GATK MarkDuplicates (v4.4.0.0; RRID:SCR_001876). Four PDX samples were excluded because of substantial mouse tissue contamination, with human‐derived reads accounting for less than 30% of total sequencing reads, and four patient samples were excluded because of low tumor purity (<10%).

### 4.3 SNV, Indel and Mutation Signature Analysis

Somatic single nucleotide variants (SNVs) and short insertion‒deletion variants (indels) were detected using Mutect2 (v4.1.2.0; RRID:SCR_026692) from GATK for both WES and WGS data. Variants were annotated using Annovar (v2020‐06‐08; RRID:SCR_012821) [89] on the RefSeq gene model, with variants in noncoding regions (upstream, downstream, intergenic, intronic, ncRNA, UTR5, UTR3, etc.) excluded. To remove potential germline variants, the remaining variants were annotated using population databases (including the 1000 Genomes, NHLBL Exome Sequencing Project (ESP6500), Genome Aggregation Database (gnomAD), Exome Aggregation Consortium), and variants with a minor allele frequency > 0.001 were excluded. To generate high‐confidence mutation calls, the following additional filtering criteria were applied: (1) significant enrichment of mutations in tumor samples compared to non‐tumor samples, as determined by Fisher's exact test; (2) at least 4 variant‐supporting reads; and (3) a Variant Allele Fraction (VAF) ≤ 0.05 in non‐tumor samples. To call somatic variants in tumor‐only samples, MuTect2 tumor‐only mode was conducted, with the following filtering criteria: (1) at least 15X coverage; (2) a minimum of 4 variant‐supporting reads; and (3) a VAF ≥ 0.1 in tumor samples. In addition, for paired PDX and patient samples, if a mutation was identified in only one sample, we rescued it in the paired sample if it was present in the raw outputs. MutSigCV (v1.4) [90] was utilized to identify significantly mutated genes, with a false discovery rate (FDR) q‐value threshold of ≤ 0.01 used to determine statistical significance.

Tumor mutation burden (TMB) was defined as the total number of somatic mutations, including base substitutions and indels, within the coding region. To minimize sampling noise, synonymous alterations were also included [91]. For the oncoprint, driver genes were selected from those included in the curated panel from Hartwig Medical Foundation (HMF) (DriverGenePanel.37.tsv, HMFtools‐Resources). Mutational signatures were extracted from high‐confidence somatic variants and categorized into single‐base substitutions (SBS), double‐base substitutions (DBS), and small insertions/deletions (INDELs) using the R packages maftools (v2.4.10; RRID:SCR_024519) [92]. Subsequently, the NMF R package (v0.28; RRID:SCR_023124) [93] was applied to perform non‐negative matrix factorization to determine the optimal number of distinct mutational signatures. The similarity of the extracted signatures to known COSMIC (v3; RRID:SCR_002260) signatures was evaluated using cosine similarity.

### 4.4 Copy Number Alterations, Tumor Purity and Ploidy Analysis

Somatic copy number alterations (CNAs) were calculated using CNVkit (v0.9.9; RRID:SCR_021917) for both WES and WGS data. For tumor‐only samples, a pooled reference was generated from multiple blood‐normal samples. Additionally, for a more detailed characterization of somatic CNAs, WES and WGS CNVkit results were independently processed by GISTIC2.0 (v2.0.23; RRID:SCR_000151). The CNA level for each gene was determined by assigning discrete values based on the output of GISTIC2.0: ‐2 for deletion, ‐1 for single‐copy loss, 0 for neutral or unchanged regions, 1 for single‐copy gain, and 2 for amplification. Driver genes located in significant GISTIC peaks, with a residual q‐value of less than 1 × 10^−^^4^, were used to generate the oncoprint plot. For purity and ploidy analysis, PURPLE (v3.7.1; RRID:SCR_022999) was used for all WGS data, utilizing a paired normal model for paired normal samples and a tumor‐only model for tumor‐only samples. For WES data, Sequenza (v3.0.0; RRID:SCR_016662) [94] was used for paired normal samples, while PureCN (v2.8.1) [95] was used for tumor‐only samples.

### 4.5 Structural Variant and Whole Genome Doubling Analysis

Structural variants (SVs) were identified using the GRIDSS‐PURPLE‐LINX suite [96], part of the HMF bioinformatics pipeline (https://github.com/hartwigmedical/hmftools). Briefly, SVs were detected by GRIDSS2 (v2.13.2; RRID:SCR_027130) [97], annotated with RepeatMasker (v4.1.2‐p1; RRID:SCR_012954) and Kraken2 (v2.1.3; RRID:SCR_026838) [98], and further filtered by GRIPSS (v2.3.5). Finally, SVs were annotated and visualized by LINX (v1.23.6; RRID:SCR_027133), which also integrated CNAs and purity results from PURPLE (v3.7.1; RRID:SCR_022999). Finally, WGD status was determined using PURPLE, with WGD identified when at least 11 autosomes exhibited a major allele copy number >1.5, as specified in the PURPLE manual.

### 4.6 Chromothripsis Analysis

Chromothripsis was identified using ShatterSeek (v0.4; RRID:SCR_026463) using recommended cutoff values [8]. Only high confidence chromothripsis calls meeting at least one of the following criteria sets were included in our analysis: (i) at least 6 interleaved intrachromosomal SVs, 7 adjacent segments oscillating between 2 copy number states, the fragment joins test, and either the chromosomal enrichment or the exponential distribution of breakpoints test; and (ii) at least 3 interleaved intrachromosomal SVs and 4 or more interchromosomal SVs, 7 adjacent segments oscillating between 2 copy number states and the fragment joins test. (iii) at least 40 intra‐ or interchromosomal SVs and the fragment joins test. Chromothripsis events were classified as canonical or noncanonical (with other complex events) based on whether more than 60% of the segments oscillated between two copy number states. To identify loss–translocation–amplification (LTA) chromothripsis events, we applied criteria adapted from a recent study [99]: focusing on the following key features: (1) biallelic TP53 disruption; (2) structural connections between chromosome 17p and other chromosome arms associated with complex genomic rearrangements (CGRs) and segmental amplification; (3) SVs involving the TP53 gene body, translocation breakpoints, or CGRs within the region between the TP53 locus and the centromere; (4) loss of the 17p terminal segment, defined as the genomic interval from the chromosome start through TP53; and (5) absence of whole‐chromosome or chromosome‐arm loss of heterozygosity (LOH). A detailed manual review of SVs involving chromosome 17 was performed using ReConPlot (v0.2).

### 4.7 ecDNA Analysis

To detect ecDNA, aligned WGS BAM files were processed by the AmpliconSuite pipeline (v1.2.2 [39], which involved downsampling the BAM files to 10x coverage, followed by copy number estimation using CNVkit (v0.9.9; RRID:SCR_021917). Genomic segments with a total copy number greater than 4.5 and a length exceeding 50 kbp were selected as candidate seed regions. The identified amplicons were then classified into four categories: ecDNA, breakage‐fusion‐bridge (BFB), complex‐non‐cyclic (CNC), and linear amplification. BFB, CNC, and linear amplicons were collectively grouped as chromosomal amplifications (ChrAmp). Patients were categorized as having ecDNA if it was detected in either their patient sample or PDX sample. CycleViz (v0.1.3) was utilized to generate circular plots of identified ecDNAs. For samples from the same patient, the GroupedAnalysisAmpSuite.py script was used to detect ecDNA, ensuring that unified seed intervals were applied across these samples. Genomic similarity among overlapping amplicons across samples was then analyzed with the amplicon_similarity.py script, which calculated similarity scores based on shared breakpoints and genomic content. These scores were compared with those from unrelated amplicons to compute P‐values, with pairs showing *p* < 0.05 designated as shared events and included in our analysis. Amplicon complexity was assessed with AmpliconClassifier scores, which incorporate both the genomic segment count and copy number diversity across all amplicon decompositions, with higher scores indicating a more structurally complex amplicon.

### 4.8 Clonal Evolution Analysis

We performed clonal analyses of SNVs and indels in 47 patient–PDX pairs for which matched germline controls were available. First, SNVs and indels were identified from matched tumor–normal WES/WGS data using Mutect2 (v4.1.2.0; RRID:SCR_026692). Allele‐specific copy number profiles were inferred using Sequenza (v3.0.0; RRID:SCR_016662) [94]. The mutation and copy number information were then converted into the input format required by PyClone‐VI (version 0.1.6; RRID:SCR_016873), a variational Bayesian framework that infers the clonal population structure of tumors by jointly modeling variant allele frequency (VAF), local copy number, and tumor purity [100]. Following the strategy described by He et al. [27], mutations were classified into clonal and subclonal categories based on PyClone‐VI clustering results: (i) if only one cluster was identified, all mutations within the cluster were considered clonal; (ii) if multiple clusters were detected, mutations in the cluster with the highest mean cancer cell fraction (CCF) were regarded as clonal. For the remaining clusters, if the mean CCF exceeded 0.9, mutations within these clusters were also considered clonal; all others were defined as subclonal. Clonal transitions between patient tumors and matched PDXs were visualized using the GgAlluvial R package (version 0.12.3) [101].

### 4.9 RNA‐seq Sequencing and Data Analysis

RNA‐seq in this study was performed primarily by Shanghai Sequanta Technologies Co., Ltd., with a subset of samples processed by Wuhan Benagen Technology Co., Ltd. For Sequanta, total RNA was extracted from ∼10 mg of frozen tissue homogenized with a mechanical grinder (Scientz) in TRIzol Reagent (Invitrogen) and cleaned up using the RNeasy MinElute Cleanup Kit (Qiagen) according to the manufacturers’ instructions. RNA integrity was assessed on an Agilent 4200 Bioanalyzer, and RNA quantity and purity were evaluated with a NanoDrop spectrophotometer. RNA with OD260/280 = 1.9–2.0 and RIN ≥ 8 was used for library construction. Stranded total RNA libraries were prepared from 100–500 ng RNA using the Illumina Stranded Total RNA Prep, Ligation with Ribo‐Zero Plus kit, which depletes cytoplasmic and mitochondrial rRNA and globin mRNA, followed by RNA fragmentation, first‐ and second‐strand cDNA synthesis (with dUTP in the second strand), AMPure XP bead purification, end repair, A‐tailing, adapter ligation, PCR amplification, and size selection to generate ∼200–300 bp inserts. Libraries were sequenced as 2 × 150 bp paired‐end reads on an Illumina NovaSeq 6000 platform (RRID:SCR_016387).

For samples processed by Wuhan Benagen, total RNA was extracted from 15–30 mg tissue using a silica‐membrane spin‐column kit (R6834 Total RNA Kit I, Solarbio) following the manufacturer's instructions. RNA quality was assessed by NanoDrop spectrophotometry, Qubit 3.0 fluorometry, and agarose gel electrophoresis. Poly(A)+ mRNA was enriched with oligo(dT) magnetic beads, fragmented, and reverse‐transcribed to generate double‐stranded cDNA. Libraries were constructed using the VAHTS Universal V6 RNA‐seq Library Prep Kit for MGI (NRM605), including end repair, A‐tailing, adapter ligation, and PCR amplification, followed by library circularization and DNA nanoball (DNB) generation. Final libraries were evaluated on an Agilent 2100 Bioanalyzer and sequenced as 2 × 150 bp paired‐end reads on a DNBSEQ‐T7 platform (RRID:SCR_017981). Adapter trimming and the removal of mouse reads from the raw sequencing data were described above. Three samples were excluded due to high levels of mouse tissue contamination. The resulting clean reads were then aligned to the human reference genome (hs37d5) using STAR (v2.7.11a; RRID:SCR_004463) [102]. Gene‐level expression, including counts, transcripts per kilobase million (TPM), and fragments per kilobase million (FPKM), was quantified using RSEM (v1.3.3; RRID:SCR_000262) [103]. Batch correction was performed using the ComBat_seq module from the sva package (v3.4.8; RRID:SCR_012836) [104]. Protein‐coding mRNAs were annotated using the GENCODE v42 gene model (gencode.v42.annotation.gtf), and the 2,000 most variable mRNA genes were selected to assess correlations between patient tumors and matched PDX samples. ImmuneScore and StromalScore were estimated from RNA‐seq data using the ESTIMATE R package (v1.0.13; RRID:SCR_026090) [105]. Differential expression analysis was conducted using the DESeq2 R package (v2.14.0; RRID:SCR_015687) [106]. In addition, gene set enrichment analysis (GSEA) was performed with the clusterProfiler R package (v4.8.1; RRID:SCR_016884) using Hallmark gene sets from MSigDB (v7.1; RRID:SCR_016863) [107, 108].

### 4.10 RNA Fusion Analysis

Fusion transcripts were detected using STAR‐Fusion (v1.13.0; RRID:SCR_025853), with false positives removed using FusionInspector, which performs supervised analysis to recover and re‐score supporting evidence for these predictions. To identify tumor‐specific fusions, fusions annotated as GTEx_recurrent, which are observed in normal samples, are excluded. We further filtered fusions by FFPM (FFPM ≤ 0.1). Additionally, for paired PDX and patient samples, if a fusion was identified in only one sample, we rescued it in the paired sample if it was present in the raw fusion pool from STAR.

### 4.11 Therapeutically Actionable Targets

The oncokb‐annotator (v3.4.1; RRID:SCR_014782) and OncoKB database were utilized to identify and annotate genes with therapeutically actionable significance [56, 57]. This resource annotates and classifies variants into distinct evidence levels, including FDA‐recognized biomarkers (Level 1), variants predicting response to standard‐of‐care therapies (Level 2), variants associated with investigational agents in clinical trials (Level 3), and variants linked to investigational agents in preliminary preclinical studies (Level 4).

### 4.12 WGBS Data Sequencing and Data Analysis

Genomic DNA was extracted from tissue samples and quantified using a Qubit fluorometer (Thermo Fisher Scientific). For each library, 200 ng of gDNA spiked with 1% unmethylated lambda DNA was sheared to ∼300 bp on a Covaris LE220R, followed by end repair and A‐tailing. Methylated adapters were ligated using the TruSeq Nano DNA HT Sample Prep Kit (Illumina). Bisulfite conversion was performed with the EZ DNA Methylation‐Gold Kit (Zymo Research) according to the manufacturer's instructions, and the resulting single‐stranded DNA was PCR‐amplified using KAPA HiFi HotStart Uracil+ ReadyMix (2×) and purified. Library concentration was measured with the Qubit dsDNA HS Assay, and fragment size distribution was assessed on an Agilent BioAnalyzer (Agilent Technologies). Qualified libraries were sequenced as 2 × 150 bp paired‐end reads on an Illumina NovaSeq 6000 platforms (RRID:SCR_016387).

Raw whole‐genome bisulfite sequencing (WGBS) reads were adapter‐trimmed and filtered to remove mouse‐derived sequences as described for genomic data preprocessing. Clean paired‐end reads were aligned to the human reference genome (hs37d5) using Bismark (v0.24.2; RRID:SCR_005604) [109] with Bowtie2 (v2.5.4; RRID:SCR_016368). PCR duplicates were removed, and methylation calls were extracted with bismark_methylation_extractor in CX context to obtain CpG, CHG, and CHH methylation. Cytosine coverage files were processed with coverage2cytosine to generate strand‐specific cytosine reports and CpG methylation profiles. Cytosines with sequencing depth <10× were excluded. For downstream analyses, methylation data were imported into methylKit (v1.24.0; RRID:SCR_005177) [110], and the 2,000 most variable CpG sites were selected to assess correlations between patient tumor and PDX samples.

### 4.13 Oxford Nanopore Long‐Read Sequencing and Data Analysis

Oxford Nanopore (ONT) Long‐read sequencing in this study was performed by Wuhan Benagen Technology Co., Ltd. High–molecular‐weight genomic DNA was extracted from tumor tissue (∼0.5 g per sample) using SDS‐based lysis and phenol–chloroform–isoamyl alcohol extraction, followed by isopropanol precipitation, 75% ethanol washes, resuspension in QIAGEN Elution Buffer, RNase A digestion, and further purification with the QIAGEN Blood & Cell Culture DNA Mini Kit (13323) according to the manufacturer's instructions. DNA quantity and purity were assessed by NanoDrop One spectrophotometry, Qubit 3.0 fluorometry, and agarose gel electrophoresis. For Oxford Nanopore long‐read sequencing, 2–2.5 µg DNA was subjected to 0.4× AMPure XP bead cleanup, quantified by Qubit, and used to prepare libraries with the Oxford Nanopore Ligation Sequencing Kit SQK‐LSK114. Briefly, DNA underwent damage repair and end repair/A‐tailing (NEBNext FFPE DNA Repair Mix and End Repair/dA‐Tailing), followed by AMPure XP cleanup and ligation of ONT adapters with Quick T4 DNA ligase. After final bead purification and preparation of the sequencing mix, libraries were loaded onto R10.4.1 flow cells and sequenced on a PromethION (RRID:SCR_017987) platform for 48–72 h, targeting ≥30× genome coverage. Raw reads were aligned to the GRCh38 reference genome using minimap2 (v2.30‐r1287; RRID:SCR_018550) with parameters “‐t 22 ‐ax map‐ont ‐MD”. For ecDNA detection, we used CoRAL [111] with the parameter –min‐bp‐support 3, using somatic copy‐number calls from CNVkit as input.

### 4.14 Treatment of PDX Model with Targeted Drug

Drug efficacy was evaluated in a KRAS p.G12C–mutant PDX model (SGH‐X‐089‐P1). When tumors reached approximately 150 mm^3^, mice were randomized into two groups (n = 7 per group): Group 1 received vehicle control (DMSO diluted in PBS), and Group 2 received Sotorasib (AMG510; Selleck, Cat# S8830) at 100 mg/kg, administered intraperitoneally twice weekly. Tumor length (L) and width (W) were measured with digital calipers every 2–3 days, and tumor volume (V) was estimated using the spheroid formula V = (L × W^2^)/2. Mice were euthanized when tumors reached approximately 1,500 mm^3^ or at the predefined experimental endpoint.

### 4.15 Immunohistochemistry and Fluorescence In Situ Hybridization

Tumor samples from PDXs and patients were collected, fixed in 4% paraformaldehyde overnight, and paraffin‐embedded. Formalin‐fixed, paraffin‐embedded (FFPE) blocks were sectioned at 4 µm, deparaffinized, and processed for hematoxylin and eosin (H&E) staining and immunohistochemistry using standard procedures. Tumor specimens were immunostained with antibodies against Ki‐67 (Abcam, Cat# ab16667, RRID: AB_302459), cleaved caspase‐3 (Cell Signaling Technology, Cat# 9661, RRID: AB_2341188), H3K36M (RevMAb Biosciences, Cat# 31‐1085‐00, RRID: AB_2716431), H3G34W (RevMAb Biosciences, Cat# 31‐1145‐00, RRID: AB_2716434), SATB2 (Servicebio, Cat# GB111449, RRID: AB_3719475), and α‐SMA (Servicebio, Cat# GB121364, RRID: AB_3683665).

Interphase FISH was performed on 4‐µm sections of FFPE tumor tissue. Slides were baked at 80°C for 30 min, deparaffinized in a heated deparaffinization solution at 68°C for 15 min, and rehydrated in 100% ethanol for 5 min at room temperature. Sections were then incubated in a preheated permeabilization solution at 90°C for 20 min, rinsed in 37°C deionized water for 3 min, and digested with a proteinase working solution at 37°C for 20 min according to the manufacturer's instructions. After digestion, slides were washed twice in 2× SSC (5 min each), dehydrated through a graded ethanol series (70%, 85%, and 100%, 2 min each), and air‐dried at room temperature. For hybridization, 10 µL of probe mixture was applied to the target area, covered with a 22 × 22 mm coverslip, and sealed to prevent evaporation. Slides were co‐denatured at 85°C for 5 min and hybridized at 42°C for 2–16 h in a hybridization chamber. All subsequent steps were performed in the dark. Coverslips were gently removed after briefly soaking slides in 2× SSC, followed by a 1‐min wash in 2× SSC at room temperature and a high‐stringency wash in 0.3% NP‐40/0.4× SSC at 68°C for 2 min. Slides were then rinsed in 37°C deionized water for 1 min, air‐dried, counterstained with 10 µL DAPI, and mounted with coverslips for fluorescence microscopy. FISH probes used in this study included SYT break‐apart (Cat# FP‐055; Wuhan HealthCare Biotechnology Co., Ltd.), EWSR1 break‐apart (Cat# FP‐051; Wuhan HealthCare Biotechnology Co., Ltd.), MDM2/CEP12 amplification (Cat# FP‐054; Wuhan HealthCare Biotechnology Co., Ltd.), MYC/CEP8 amplification (Cat# FP‐015; Wuhan HealthCare Biotechnology Co., Ltd.), and CCNE1/19p13 (Cat# FP‐360; Wuhan HealthCare Biotechnology Co., Ltd.), as well as NFIB/CEN9 probes (Cat# TLB301299; Biolancet, Beijing, China).

For metaphase FISH, dissociated PDX tumor cells were treated overnight with 0.02‐0.1 µg/mL colchicine (Beyotime, Cat# ST1173‐1g), washed once with PBS, and incubated in 75 mmol/L KCl at 37°C for 30 min. Cells were then fixed in methanol:glacial acetic acid (3:1, v/v) with three changes and dropped onto glass slides. After drying overnight, slides were equilibrated in 2× SSC, dehydrated through 70%, 85%, and 100% ethanol, denatured at 75°C for 2 min, and hybridized with FISH probes at 37°C overnight. Slides were washed twice in 0.4× SSC containing 0.3% IGEPAL CA‐630 and once in 2× SSC containing 0.1% IGEPAL CA‐630, counterstained with DAPI, and mounted in antifade medium. FISH images were acquired using an OLYMPUS BX43 fluorescence microscope (Olympus, Tokyo, Japan). For karyotype analysis, metaphase spreads prepared from the same colchicine‐treated cultures were Giemsa–Trypsin (GTG) banded and analyzed according to standard cytogenetic criteria.

### 4.16 Statistical Analysis

All statistical analyses were performed in R (v4.3.0; RRID:SCR_001905). Statistical methods are indicated in the corresponding figure legends. Kaplan‐Meier survival curves and log‐rank tests were performed using the R package ‘survminer’ (v0.4.9; RRID:SCR_021094). Overall survival was defined as the interval from the date of initial diagnosis to death from any cause or last follow‐up.

## Author Contributions

**Weisong Zhao**: investigation, methodology, validation, visualization, data curation, formal analysis, writing – review and editing, writing – original draft, software, resources. **Zhiwei Cheng**: investigation, methodology, validation, visualization, formal analysis, software, data curation, resources, writing – original draft. **Kai Tian**: validation, investigation, writing – review and editing, writing – original draft, formal analysis, resources, data curation. **Mengxiong Sun**: investigation, writing – review and editing, resources, data curation, validation, formal analysis. **Zhuoying Wang**: investigation, writing – review and editing, project administration, resources. **Ming Jiao**: writing – review and editing, investigation, resources. **Jens Luebeck**: investigation, methodology, software, writing – review and editing. **Kaiyuan Zhu**: investigation, writing – review and editing, methodology, software. **Xiao Zhang**: investigation, writing – review and editing, resources. **Dongqing Zuo**: investigation, writing – review and editing, resources. **Hongsheng Wang**: investigation, writing – review and editing, resources. **Chongren Wang**: investigation, writing – review and editing, resources. **Jiakang Shen**: investigation, writing – review and editing, resources. **Xiaojun Ma**: investigation, writing – review and editing, resources. **Gangyang Wang**: investigation, writing – review and editing, resources. **Zongyi Wang**: investigation, writing – review and editing, resources. **Jing Han**: investigation, writing – review and editing, resources. **Kaiyuan Liu**: investigation, writing – review and editing, resources. **Haoran Mu**: investigation, writing – review and editing, resources. **Weiqi Ma**: investigation, writing – review and editing, resources. **Yongyong Ren**: investigation, writing – review and editing, resources. **Shibing Guo**: investigation, writing – review and editing, resources. **Huangxin Tu**: investigation, writing – review and editing, resources. **Lingfeng Yu**: investigation, writing – review and editing, resources. **Yining Tao**: investigation, writing – review and editing, resources. **Yu Lv**: investigation, resources, writing – review and editing. **Hui Lu**: investigation, methodology, writing – review and editing. **Owen S. Chapman**: investigation, methodology, writing – review and editing. **Rishaan Kenkre**: investigation, writing – review and editing, methodology. **Lukas Chavez**: investigation, writing – review and editing, methodology. **Zhengdong Cai**: writing – review and editing, resources, supervision, funding acquisition. **Yuqin Yang**: investigation, writing – original draft, writing – review and editing, resources, supervision, conceptualization. **Wei Sun**: conceptualization, writing – original draft, methodology, resources, writing – review and editing, investigation, supervision. **Yafei Jiang**: conceptualization, methodology, resources, writing – review and editing, writing – original draft, investigation, supervision, funding acquisition. **Yingqi Hua**: conceptualization, methodology, writing – review and editing, writing – original draft, resources, funding acquisition, investigation, supervision.

## Conflicts of Interest

The authors declare no conflicts of interest.

## Supporting information




**Supporting File 1**: advs78200‐sup‐0001‐SuppMat.docx.

**Supporting File 2**: advs78200‐sup‐0002‐SuppMat.xlsx.

## Data Availability

WES and RNA‐seq data for a subset of patients are available in the Genome Sequence Archive for Human (GSA; https://ngdc.cncb.ac.cn/gsa‐human/) under accession number HRA003260. RNA‐seq data for 88 patients in the TARGET‐OS cohort and 265 patients in the TCGA‐Sarcoma cohort were downloaded from the UCSC Xena website (https://xena.ucsc.edu/). Newly generated sequencing data from this study have been deposited in GSA‐Human under accession number HRA018726. Access will be provided through the GSA data access committee; additional inquiries can be directed to Dr. Yingqi Hua (yhua@shsmu.edu.cn). PDX models used in this study are also available from Dr. Yingqi Hua upon reasonable request.
