# Supplementary material for: A Living Multi‐Omics PDX Biobank of High‐Risk Sarcomas Reveals ecDNA‐Driven Oncogene Amplification in Osteosarcoma and Enables Preclinical Precision Oncology
Source: Adv Sci (Weinh). 2026 Oct 12:e78200. Online ahead of print. doi: 10.1002/advs.78200 (PMC13658889; doi:10.1002/advs.78200)
Supplement: Supplementary file 1 [file ADVS-9999-e78200-s001.docx]

**Supplementary Figure S1. Clinical characteristics of all enrolled patients and overview of longitudinal PDX models.**

**Supplementary Figure S2. Genomic landscape of osteosarcoma versus other sarcoma PDX models.**

**Supplementary Figure S3. Chromothripsis events in WGS-profiled PDX models.**

**Supplementary Figure S4. Gene fusion landscape in the SGH sarcoma PDX cohort and representative examples of diagnostically reclassified PDX models.**

**Supplementary Figure S5. High-level ectopic amplifications observed in two karyotypically complex PDX models.**

**Supplementary Figure S6. ecDNA amplification in paired primary and recurrent osteosarcoma PDX models.**

**Supplementary Figure S7. Amplicon status switching between ChrAmp and ecDNA in patient–PDX pairs.**

**Supplementary Figure S8. Paired amplicon features in patient tumors and matched PDX models, and characterization of an *NFIB* ecDNA amplicon.**

**Supplementary Figure S9. Multi-omics concordance between patient tumors and matched PDX models.**

**Supplementary Figure S10. Clonal evolution dynamics from patient tumors to PDX models.**

**Supplementary Figure S1. Clinical characteristics of all enrolled patients and overview of longitudinal PDX models. A,** Distribution of tumor histotypes among all patients whose tumors were subjected to PDX engraftment. **B,** Demographic and clinical characteristics of these patients, including sex, age group, anatomic tumor site, specimen type (resection vs biopsy), and disease status (primary, metastatic, or recurrent). **C,** Longitudinal PDX models and sequencing information. Left, swimmer plot showing the timing and source of PDX derivation (biopsy, primary, relapse, or metastasis) relative to years from diagnosis for each patient. Right, overview of sequencing information for these longitudinal PDX models. **D,** Hallmark gene set enrichment analysis (GSEA) comparing osteosarcoma tumors from patients with successful (n = 30) versus failed (n = 28) PDX engraftment. The bar plot summarizes the top five upregulated and top five downregulated Hallmark pathways ranked by absolute normalized enrichment score (NES); middle and right panels show enrichment plots for HALLMARK_MYC_TARGETS_V1 and HALLMARK_MYC_TARGETS_V2, respectively.

**Supplementary Figure S2. Genomic landscape of osteosarcoma versus other sarcoma PDX models. A,** Clinical and genomic annotations for each of 148 unique PDX models, stratified by osteosarcoma (left) and other sarcoma (right); each column represents one PDX. **B,** Recurrently mutated cancer genes in osteosarcoma versus other sarcoma PDX models, with genes displayed in the same order as in Figure 2B; colors indicate mutation class (missense, nonsense, frameshift, in-frame, or multi-hit). **C,** Relative contributions of inferred mutational signatures for each PDX, shown separately for osteosarcoma and other sarcoma. **D,** Somatic copy-number alterations affecting driver genes in osteosarcoma versus other sarcoma PDX models, with genes displayed in the same order as in Figure 2C; copy-number states are shown as high-level amplification (+2), low-level gain (+1), neutral (0), shallow loss (−1), and deep deletion (−2).

**Supplementary Figure S3. Chromothripsis events in WGS-profiled PDX models. A,** Proportion of WGS-sequenced PDX samples with or without chromothripsis. **B,** Frequency of chromothripsis in osteosarcoma versus other sarcoma PDX models. **C,** Percentage of chromosomes affected by chromothripsis events. **D–F,** Representative examples of LTA chromothripsis in three osteosarcoma PDX models, showing rearrangement and copy-number profiles for the affected chromosomes visualized with ReConPlot. DEL, deletion-like rearrangement; DUP, duplication-like rearrangement; h2hINV, head-to-head inversion; t2tINV, tail-to-tail inversion.

**Supplementary Figure S4. Gene fusion landscape in the SGH sarcoma PDX cohort and representative examples of diagnostically reclassified PDX models. A,** Total number of fusion events in osteosarcoma (n = 106 PDXs) and other sarcoma PDX models (n = 37). **B,** Fusion burden per sample in osteosarcoma versus other sarcomas, shown as the number of fusion events per tumor; P value (Wilcoxon rank-sum tests) is indicated. **C,** Representative H&E and *EWSR1* break-apart FISH images from patient tumor and matched PDX (SGH-X-122), 50 μm for H&E and 10 μm for FISH. **D,** Somatic SV and copy-number profiles for chromosomes 11 and 22 in SGH-X-122-P1. **E,** Circos plot of SGH-X-122-P1 showing genome-wide somatic copy-number changes and SVs. **F,** Rearrangement and copy-number profiles demonstrating *EWSR1-FLI1* fusion in one Ewing sarcoma PDX model.

**Supplementary Figure S5. High-level ectopic amplifications observed in two karyotypically complex PDX models.** **A–B**, SV views of highly complex ecDNA amplicons in the patient tumor SGH-099-D (A) and its matched PDX SGH-X-111-P1 (B). **C–D**, Interphase (C) and metaphase (D) *CDK4/CEN12* (left) and *MYC/CEN8* (right) DNA FISH in the patient tumor SGH-099-D and its matched PDX SGH-X-111-P1. Scale bars, 10 μm. **E–G**, SV views of *MYC*-bearing amplicons in three osteosarcoma PDXs: SGH-X-140-P1 (E; linear amplification), SGH-X-141-P0 (F; complex non-cyclic amplification), and SGH-X-142-P0 (G; ecDNA). H, Interphase (left) and metaphase (right) *MYC/CEN8* DNA FISH in SGH-X-142-P0. Scale bars, 10 μm. I–J, G-banded karyotypes from PDXs SGH-X-111 (I) and SGH-X-142 (J). Arrows mark structurally abnormal chromosomes, and “mar” denotes an unidentifiable marker chromosome. All AmpliconSuite analyses were performed using full-coverage BAM files.

**Supplementary Figure S6. ecDNA amplification in paired primary and recurrent osteosarcoma PDX models. A,** WES-derived log₂ copy-number profiles for chromosome 8 in the primary patient tumor and matched PDX samples; the red line marks the *MYC* locus. **B,** SV view of the MYC-bearing ecDNA amplicon in the primary patient tumor SGH-027-D. **C,** SV view of the CCNE1-bearing ecDNA amplicon in the matched primary PDX SGH-X-033-P4. **D,** Interphase *CCNE1/CEN19* DNA FISH in primary (left) and recurrent (right) PDX samples; arrows indicate nuclei with different levels of *CCNE1* amplification. Scale bars, 10 μm. **E** SV view of a *CCNE1*-bearing ecDNA amplicon in the primary PDX, reconstructed with CoRAL from long-read sequencing data. **F–G,** SV view of complex *MYC*-bearing ecDNA amplicons in primary (F) and recurrent (G) PDX samples

**Supplementary Figure S7. Amplicon status switching between ChrAmp and ecDNA in patient–PDX pairs. A–C,** Patient–PDX pairs in which an amplicon is classified as ChrAmp in the patient tumor but as ecDNA in the matched PDX (top, patient; bottom, PDX), with amplicon complexity and similarity scores shown. **D,** Example of an ecDNA amplicon present at low copy number in the patient tumor that shows markedly increased copy number in the matched PDX while retaining a highly similar structure.

**Supplementary Figure S8. Paired amplicon features in patient tumors and matched PDX models, and characterization of an *NFIB* ecDNA amplicon. A,** Box plots comparing amplicon size, copy number, and structural complexity of paired amplicons between patient tumors and matched PDXs, shown separately for ecDNA and ChrAmp; “ns” indicates *p* ≥ 0.05 by paired Wilcoxon rank-sum tests. **B,** GTG-banded karyotype (left) and metaphase spread (right) of the PDX SGH-X-130; arrows mark structurally abnormal chromosomes, and “mar” denotes unidentifiable marker chromosomes. **C,** SV view of the *NFIB*-bearing ecDNA amplicon on chromosome 9, reconstructed with CoRAL from long-read sequencing data. **D,** CoRAL-derived reconstruction of a single NFIB ecDNA cycle and its inferred copy number.

**Supplementary Figure S9. Multi-omics concordance between patient tumors and matched PDX models. A,** Hallmark GSEA of paired RNA-seq profiles, showing top pathways upregulated (Activated) and downregulated (Suppressed) in PDXs. **B,** Spearman correlation heatmap of RNA-seq profiles from multi-passage PDX samples based on the top 2,000 most variable protein-coding genes; annotation bars indicate tumor type. **C,** Spearman correlation heatmap of gene-level copy-number profiles for matched patient–PDX pairs with tumor purity ≥30%. **D,** Venn diagrams showing overlap of all somatic mutations (left) and driver mutations (right) between patient tumors and PDXs. **E,** Driver mutation concordance per pair, showing fractions of shared, patient-unique, and PDX-unique driver mutations; the dashed line indicates the median shared fraction. **F,** Spearman correlation heatmap of CpG methylation profiles from WGBS data for matched pairs, based on the 2,000 most variable CpG sites. **G,** Comparison of SV burden between patient tumors and matched PDXs; each line connects a pair and the p-value is calculated using the paired Wilcoxon signed-rank test.

**Supplementary Figure S10. Clonal evolution dynamics from patient tumors to PDX models.**

**A,** Alluvial diagram depicting the flow of mutation clonality from patient tumor (PT) to PDX for 47 paired samples. Clonal mutations are shown in red and subclonal mutations in blue; band width reflects the number of mutations. **B–D,** Alluvial diagrams illustrating three evolutionary patterns across all analyzed patient-PDX pairs: (B) clone sweeping, (C) branch seeding, and (D) clone retention. PT, patient tumor; PDX, patient-derived xenograft.
